# Microfluidic‐Based Reconstitution of Functional Lymphatic Microvasculature: Elucidating the Role of Lymphatics in Health and Disease

**DOI:** 10.1002/advs.202302903

**Published:** 2023-12-07

**Authors:** Jean C. Serrano, Mark R. Gillrie, Ran Li, Sarah H. Ishamuddin, Emad Moeendarbary, Roger D. Kamm

**Affiliations:** ^1^ Department of Mechanical Engineering Massachusetts Institute of Technology Cambridge MA 02139 USA; ^2^ Department of Biological Engineering Massachusetts Institute of Technology Cambridge MA 02139 USA; ^3^ Department of Medicine University of Calgary Calgary AB T2N 1N4 Canada; ^4^ Center for Systems Biology Massachusetts General Hospital Research Institute Boston MA 02114 USA; ^5^ Department of Mechanical Engineering University College London Torrington Place London WC1E 7JE UK; ^6^ 199 Biotechnologies Ltd Gloucester Road London W2 6LD UK

**Keywords:** immune cells, lymphatics, microfluidics, tissue engineering, transport phenomena

## Abstract

The knowledge of the blood microvasculature and its functional role in health and disease has grown significantly attributable to decades of research and numerous advances in cell biology and tissue engineering; however, the lymphatics (the secondary vascular system) has not garnered similar attention, in part due to a lack of relevant in vitro models that mimic its pathophysiological functions. Here, a microfluidic‐based approach is adopted to achieve precise control over the biological transport of growth factors and interstitial flow that drive the in vivo growth of lymphatic capillaries (lymphangiogenesis). The engineered on‐chip lymphatics with in vivo‐like morphology exhibit tissue‐scale functionality with drainage rates of interstitial proteins and molecules comparable to in vivo standards. Computational and scaling analyses of the underlying transport phenomena elucidate the critical role of the three‐dimensional geometry and lymphatic endothelium in recapitulating physiological drainage. Finally, the engineered on‐chip lymphatics enabled studies of lymphatic‐immune interactions that revealed inflammation‐driven responses by the lymphatics to recruit immune cells via chemotactic signals similar to in vivo, pathological events. This on‐chip lymphatics platform permits the interrogation of various lymphatic biological functions, as well as screening of lymphatic‐based therapies such as interstitial absorption of protein therapeutics and lymphatic immunomodulation for cancer therapy.

## Graphical abstract

By implementing microfluidic techniques, researchers successfully mimic in vivo lymphatic growth and function on‐chip. This breakthrough permits detailed study on lymphatic physiological drainage, and mechanistic insights into lymphatic‐immune interactions, thereby enabling advanced screening of lymphatic‐based therapeutics.

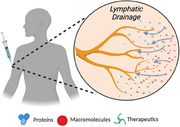

## 1 Introduction

Most human tissues contain a secondary vascular system known as the lymphatics comparable in complexity to their blood vasculature. Both systems serve as an elaborate, hierarchal network of vessels lined by endothelial cells that serve as conduits for fluid, protein, and cellular transport.^[^ ^1^ ^]^ However, in contrast to the blood vascular system where fluid is continuously recirculating through different tissues, the lymphatic system operates as a one‐way transport pathway that collects fluid, proteins, and cells from the interstitial space of tissues and returns them to the systemic circulation.^[^ ^2^ ^]^ Under this mechanism, lymphatics contribute to tissue fluid and osmotic pressure homeostasis. In addition, the lymphatic system acts as an immune checkpoint by transporting antigen and antigen‐presenting immune cells from the interstitial tissue to the lymph nodes, where resident immune cells respond to localized or systemic inflammation and infection.^[^ ^3^ ^]^

While lymphatics are critical for physiological transport, research toward understanding their biological function\interactions and pathological alterations has been severely lacking, especially when compared to the vast literature on the blood vasculature.^[^ ^4^ ^]^ This situation has begun to change, though, in recognition of the central role of the lymphatic circulation in human immune response, in both the progression and therapeutic control of an ever‐increasing array of pathologies. One of the overriding challenges in lymphatics‐focused studies has been the lack of appropriate and robust experimental models for the interrogation of biological mechanisms implicated in lymphatic development, physiology, and disease. For years, animal models served as the gold standard to evaluate the biological function of lymphatics, as well as its implications in pathological events such as inflammation, immune cell trafficking, pathogen response, and cancer progression.^[^ ^5^, ^6^, ^7^ ^]^ Despite fully recapitulating physiological responses, animal studies offer limited control over local environmental cues, present barriers to isolate and describe the direct and indirect systemic effects of the modulated parameters, and their findings are often difficult to relate to corresponding phenomena in humans due to species differences. To mitigate these limitations, numerous groups have implemented in vitro models to perform reductionist studies on lymphatic development and pathogenesis with the ability to isolate the individual contribution of regulated cues in the cellular microenvironment.^[^ ^8^, ^9^, ^10^, ^11^ ^]^ However, the simplicity of such models often leads to a lack of physiological relevance, limiting their applicability to study in vivo events.

**Figure 1:**
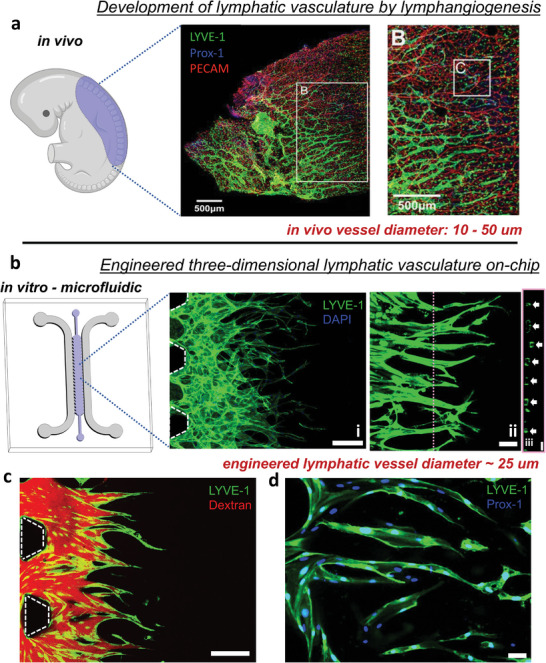
Recapitulation of in vivo‐like lymphatic capillaries by the microfluidic model of lymphangiogenesis. a) In vivo embryonic lymphangiogenesis in the dorsal skin section of a mouse embryo. Confocal images correspond to a whole‐mount anterior dorsal skin (blue region in the schematic diagram) with PROX‐1 (blue) and LYVE‐1 (green) lymphatic specific markers, and blood vascular marker PECAM1 (red). Scale bars are specified in each image. Reproduced with permission via CCC.^[^ ^27^ ^]^ b) Microfluidic‐based in vitro model of lymphangiogenesis, the blue region highlights the ECM channel compartment, and gray regions depict fluidic channels. Representative images of lymphatic vessels developed within the microfluidic system (i), a focused view of the engineered lymphatic vessels (ii), and a corresponding orthogonal view (iii) depicting lumen compartments. White arrows indicate vascular lumens. Scale bars are 200, 50, and 25 µm, respectively. c) Intravascular fluorescent‐dextran (red) indicating patency of the engineered lymphatic microvasculature (green). Scale bar is 200 µm. d) On‐chip lymphatic vessels stained for PROX‐1, lymphatic transcriptional factor (blue). Scale bar is 50 µm.

Given these limitations, there is a need for next‐generation in vitro platforms with enhanced recapitulation of the complexities of the in vivo microenvironment, while leveraging tight control over the biological interactions under study. This has driven the development of microfluidic technologies that address these needs by the culture of human‐sourced cells in three‐dimensional (3D) environments that mimic tissue architecture with precise manipulation of the cellular microenvironment.^[^ ^12^, ^13^, ^14^ ^]^ However, studies implementing microfluidic systems to generate lymphatic vasculature are exceedingly scarce. Apart from simple monolayer systems that lack anatomical resemblance, relatively few published works have attempted to recreate lymphatic vessels on‐chip.^[^ ^14^, ^15^, ^16^ ^]^ Most of these studies implement a single lymphatic capillary on‐chip system to study solute permeability or paracrine signaling/conditioning between lymphatic and fibroblast/tumor cells.^[^ ^17^, ^18^, ^19^, ^20^ ^]^ While such studies have provided fundamental insight into the biology of lymphatics, they have limited utility to study lymphatic function since a simple, single‐capillary structure fails to recapitulate the branching hierarchical lymphatic structure found in vivo (**Figure** **1**a). Thus, a more appropriate system would integrate the natural self‐assembly of lymphatics to fully reconstitute a vascular bed that permits the functional evaluation of lymphatic physiology on a tissue‐relevant scale.

As with most tissues and organs, the unique patterning of lymphatic vascular structures arises from the precise spatiotemporal control of biomolecules and biomechanical stimuli which guide migration, growth, and remodeling of cells during development.^[^ ^21^ ^]^ By emulating the distribution and transport of these signals, on‐chip systems can be further exploited to engineer microvasculature that inherently captures its native, complex network structure via vascular self‐assembly.^[^ ^22^ ^]^ In recent crucial studies, microfluidic devices were successfully implemented to induce lymphatic angiogenesis (lymphangiogenesis) under this approach.^[^ ^14^, ^23^ ^]^ However, mass transport not only plays a critical role in shaping the distribution of factors that give rise to these vascular structures but also constitutes the fundamental phenomena by which lymphatic drainage and lymphatic‐immune biochemical signaling occur.^[^ ^24^ ^]^ To the best of our knowledge, no published work has provided an on‐chip system that fully reconstitutes tissue‐scale lymphatic vascular formation with relevant microenvironmental cues for physio‐/pathological responses, adaptable for biological interrogation and preclinical drug assessments.

Aiming to address these shortcomings, we designed a microfluidic approach to generate functional lymphatic microvascular networks in a 3D hydrogel compartment (Figure 1b–d). We screened for the optimal balance of growth factors, and interstitial fluid flow to induce controlled levels of angiogenic sprouting by lymphatic endothelial cells and achieve in vivo‐like lymphatic vessel morphology. Subsequently, we quantified the tissue drainage functionality of our engineered lymphatic microvasculature by which we validated solute drainage rates comparable to in vivo measurements. We also analyzed the underlying transport phenomena, elucidating the importance of a 3D geometry and the lymphatic endothelium to recapitulate physiological drainage. Finally, we demonstrate the utility of our on‐chip engineered lymphatics to study lymphatic‐immune interactions, specifically during inflammatory responses corresponding to an increased number of immune cells recruited to the lymphatics, guided by chemical gradients of lymphatic‐secreted chemokines.

## 2 Results and Discussion

### 2.1 Engineering Physiological Lymphatic Microvasculature via Biochemical or Biomechanical Stimuli

**Figure 2:**
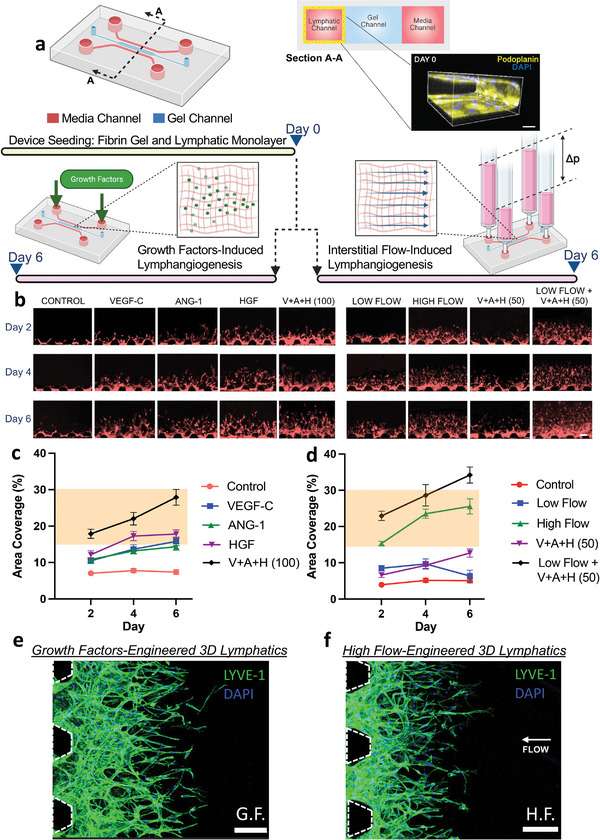
Tissue‐scale reconstitution of lymphatic microvasculature via on‐chip application of controlled biochemical and/or biomechanical cues. a) Schematic of the microfluidic device implemented for compartmentalized cell culture and precise delivery of biomolecules and fluid transport. Cross‐sectional view of the microfluidic device. Confocal projection of a lymphatic monolayer seeded at the media channel with podoplanin staining the cell membrane (yellow) and DAPI staining the nuclei (blue). Scale bar is 100 µm. b) Representative images of lymphatic sprouting for experimental conditions corresponding to stimulus by growth factors and/or interstitial flow. Red‐fluorescence of each image corresponds to RFP expressed by the lymphatic endothelial cells. Scale bars are 100 µm. Quantitative analysis of lymphatic microvascular area of coverage under biochemical stimulus with growth factors c) and biomechanical stimulus by high or low interstitial flow d). Data shown corresponds to the mean ± S.E.M., *n* = 3 samples (microfluidic devices) per condition, and *m* = 9 ROI images per sample. Highlighted regions correspond to in vivo values. Tissue‐scale lymphatic vasculature grown via e) growth factors (G.F.): VEGF‐C, ANG‐1, and HGF or f) high interstitial fluid flow (≈5 µm s^−1^) (ii, H.F.). Confocal images depict LYVE‐1 and DAPI staining as green and blue, respectively. Scale bars are 200 µm.

To engineer physiologically‐functional, human lymphatic microvasculature, we implemented a microfluidic platform that facilitated precise control over the biochemical and biophysical factors native to the development of the lymphatics, and their physiological microenvironment. As such, the microfluidic device (**Figure** **2**a) facilitated the compartmentalization of culture media, extracellular matrix (ECM), and cells within a 3D environment. Additionally, the PDMS‐based device allowed for high‐resolution imaging of microscale, cellular events via confocal microscopy. We first characterized the diffusive transport of a 70 kDa FITC‐dextran through the ECM by measuring the fluorescence intensity profile across the gel channel 120 min after introducing the fluorophore to the media channel (Figure S1a, Supporting Information). The resulting profile was fitted to the 1D unsteady solution of Fick's Second Law from which the effective diffusion coefficient (*D*) of ≈45 µm^2^ s^−1^ was obtained that is within the range of values measured *ex vivo* for the same fluorescent tracer in tissues.^[^ ^25^, ^26^ ^]^ Using this computed diffusion coefficient, we estimated the timescale required for diffusion of growth factors across the gel region as ≈*w^2^/D* (with *w* the width of the gel channel) to be ≈2 hrs, which is a small fraction of the several days required for lymphatic vascular growth. Furthermore, this time scale (calculated for a 70 kDa‐sized molecule with comparable size to various soluble proteins) is sufficiently short to ensure the adequate delivery of growth factors.

In addition to the diffusive transport of growth factors, vascular fluid flow drives the mass transfer of biomolecules and proteins throughout tissues.^[^ ^28^ ^]^ This convective transport of fluid through tissue ECM (interstitial flow) and into the lymphatics facilitates the percolation and drainage of extracellular fluid and solutes via the lymphatics to maintain hydrostatic and oncotic pressure homeostasis in tissues.^[^ ^29^ ^]^ Our microfluidic platform enables us to mimic this convective transport by introducing a hydrostatic pressure imbalance between media channels (Figure 2a), which drives flow across the compartmentalized ECM region. Furthermore, we measured the interstitial fluid velocity as a function of the hydrostatic pressure difference and thereby calculated the hydraulic permeability of the fibrin‐based ECM (Figure S1b, Supporting Information). We determined the pressure differences needed to drive interstitial fluid flow at ≈1 µm s^−1^ representing physiological/homeostatic conditions^[^ ^30^ ^]^ or, at higher velocities ≈4 µm s^−1^ relevant to an inflamed tissue and tumor microenvironments.^[^ ^31^, ^32^ ^]^

Control over the biological transport of lymphangiogenic factors allowed us to screen for the optimal conditions to grow 3D lymphatic capillaries resembling their native, in vivo morphology (Figure 2a). Starting from a confluent monolayer of human lymphatic endothelial cells at the media‐gel interface, growth factors previously identified as key mediators of developmental lymphangiogenesis (VEGF‐C,^[^ ^33^ ^]^ ANG‐1,^[^ ^34^ ^]^ HGF^[^ ^35^ ^]^) were introduced into the opposite media channel to generate a localized source of factors, that would steadily diffuse toward the lymphatic monolayer, resulting in lymphatic sprouting into the central ECM compartment. The formation of lymphatic vessels was imaged for 6 days (Figure 2b), during which morphological properties such as vessel diameter and lymphatic area of coverage were quantified. From this characterization, we identified conditions relevant to the vascular morphological values reported in vivo, such as lymphatic vessel diameters of 10–50 µm,^[^ ^36^ ^]^ and lymphatic projected area coverage of 15–30%.^[^ ^37^, ^38^, ^39^, ^40^, ^41^ ^]^ The addition of lymphangiogenic growth factors, individually or in combination, consistently resulted in lymphatic sprouts with diameters well‐within in vivo values (Figure S2a, Supporting Information). However, only the simultaneous addition of all three growth factors (100 ng mL^−1^ each) led to the full range of values corresponding to in vivo lymphatic vascular coverage area (Figure 2c,e). We also performed additional experiments with dual combinations of the growth factors (Figure S3), from which we concluded that only the application of all three growth factors gives rise to physiological 3D lymphatic vasculature.

In an alternate approach, we introduced interstitial flow to stimulate the growth of lymphatic capillaries based on previous work showing that sprouting is induced against the direction of interstitial flow as it passes from the extracellular space toward the vascular compartment.^[^ ^14^, ^42^ ^]^ We considered two interstitial flow velocities: a low flow (LF) regime corresponding to homeostatic, physiological conditions (≈1 µm s^−1^) and a high flow (HF) regime of pathological nature (3–6 µm s^−1^).^[^ ^30^, ^31^, ^32^ ^]^ Both LF and HF elicited lymphatic sprouting during the initial days of culture, however, the extent of vascular invasion was significantly greater under HF conditions (Figure 2b,d) with HF matching more closely the area of coverage found in vivo. Thus, biomechanical stimulus, imparted by pathological levels of interstitial flow, can be exploited as a means to generate tissue‐scale lymphatic vasculature (Figure 2d). This finding is in line with in vivo pathological microenvironments (i.e., a developing tumor or an inflamed wound site) where a buildup of interstitial fluid pressure, from leaky blood vessels, leads to higher interstitial fluid flow toward the lymphatics, thus evoking lymphangiogenesis.^[^ ^43^ ^]^ We also considered the synergistic effects of biochemical and biomechanical stimuli using a lower dose (50 ng mL^−1^) of lymphangiogenic growth factors coupled with low interstitial flow (≈1 µm s^−1^). Interestingly, this approach led to excessive lymphangiogenic activity as indicated by hyper‐physiological values for the area coverage (Figure 2b). Moreover, all the diameters remained within the range of in vivo values (Figure S2b, Supporting Information), confirming the advantages of this approach for growing lymphatic capillaries with an appropriate length scale. We thus identified high interstitial flow, by itself, as the optimal biomechanical stimulus to achieve physiologically relevant lymphatic microvasculature and used this in all subsequent experiments. It is worth mentioning, however, that alternate conditions could be further iterated to engineer lymphatic structures with in vivo morphological properties. Of special interest are the concurrent stimuli of growth factors, shear stress, and matrix stiffness given their extensive interplay during the native formation and regeneration of lymphatic structures.^[^ ^44^, ^45^ ^]^

Furthermore, we also observed blind/blunt‐ended, small‐scale (≈25 µm), 3D, and lumenized structures in our engineered vasculature (Figure 1b,c and Figure 2e,f). Subsequent immunostaining affirmed the expression of lymphatic‐specific markers (Figure 1d) such as lymphatic vascular endothelial receptor‐1 (LYVE‐1),^[^ ^46^ ^]^ and the upregulation of transcriptional factor PROX‐1^[^ ^47^ ^]^ each of which is indicative of in vivo lymphatic features. In assessing the physiological significance of our vascular platform, it is imperative to underscore the urgent demand for quantitative assays that extend beyond the limited metrics offered by traditional morphological evaluations. Complementing this, functional assays and advanced data analyses are set to enhance the granularity of in vitro data interpretation and its correlation to the in vivo lymphatic system.^[^ ^48^, ^49^, ^50^ ^]^ Building upon this effort, we continue our study with significant detail into characterizing the physiological functionality of our engineered lymphatics across health and disease scenarios.

### 2.2 Engineered Lymphatics Exhibit Solute Drainage Rates Comparable to In Vivo

**Figure 3:**
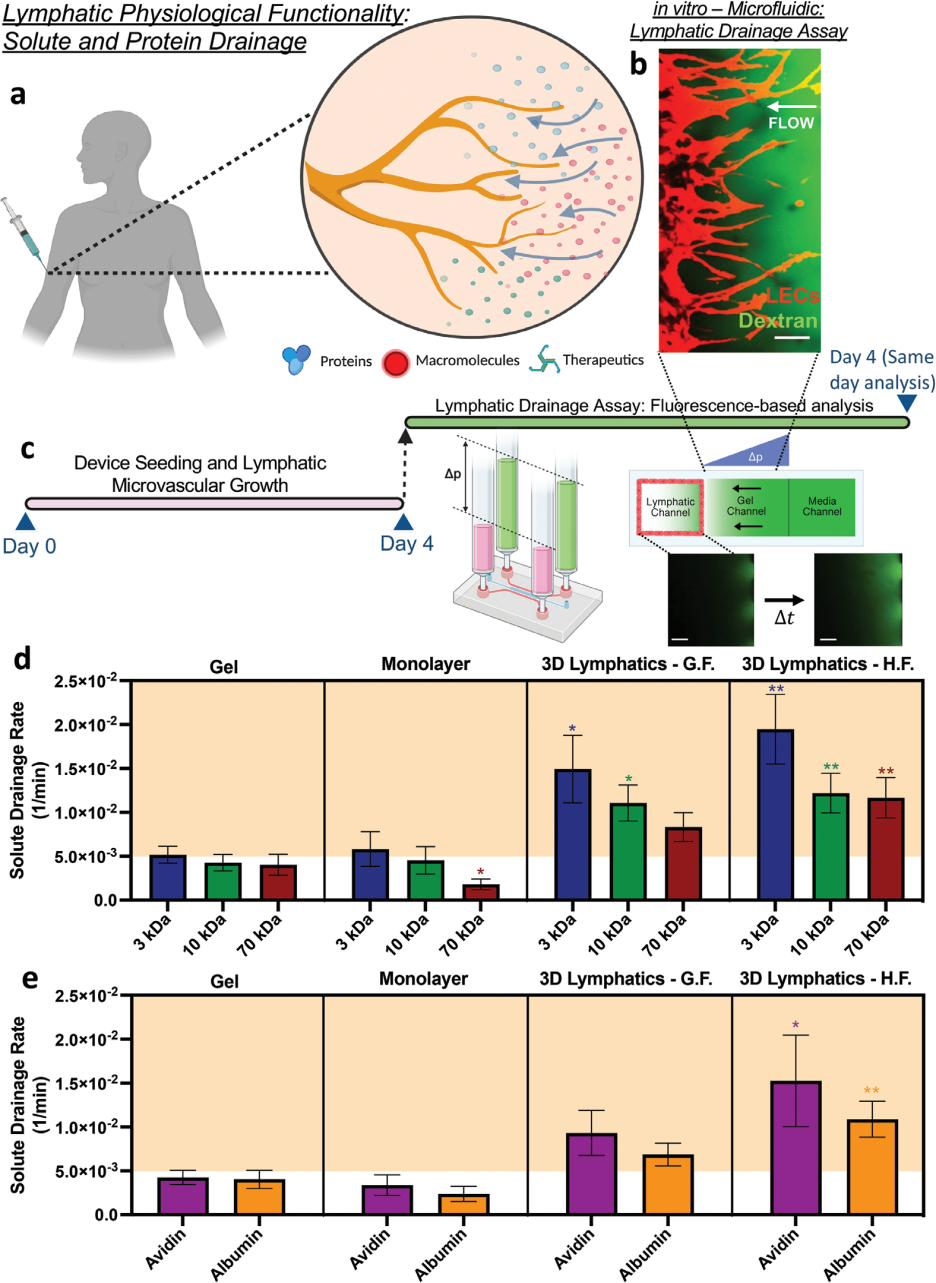
On‐chip lymphatics recapitulate physiological solute and protein drainage. a) Schematic depicting physiological drainage by lymphatics in vivo. b) Confocal image of lymphatic drainage in our microfluidic system showing the lymphatic network (RFP expression (red)) and the fluorescent dextran tracer (green) flowing right‐to‐left. Scale bar is 100 µm. c) Protocol to evaluate drainage rates of our microfluidic system where a hydraulic pressure difference induces physiological flow toward the lymphatics as the increase of fluorescence signal at the lymphatic media channel is measured over time. d,e) Quantification of solute drainage rates at an IF speed of ≈1 µm s^−1^ of dextrans with varying molecular weight (d) or two representative biological molecules (e) for different experimental settings: bare gel (devoid of cells), lymphatic monolayer (formed on left gel face), 3D lymphatics formed with growth factors (GF), and 3D lymphatics formed with high interstitial flow (HF). Data shown corresponds to the mean ± S.E.M., *n* = 2–4 samples (microfluidic devices) per condition, and *m* = 3 ROI images per sample. Statistical significance is reported with respect to the same molecular weight tracer/protein (color‐guided) in the gel‐only system, where *p < 0.05, **p < 0.01 (based on the two‐tailed t‐test). Highlighted regions correspond to in vivo values.

The lymphatic system serves an integral role in maintaining tissue homeostasis by clearing excess fluid, plasma proteins, pathogenic agents (antigens), and endo‐/exogenous carriers (vesicles/exosomes, therapeutics) from the peripheral tissues into the systemic circulation^[^ ^29^ ^]^ (**Figure** **3**a). Physiologically, these factors are exchanged between the blood vasculature and the tissue interstitium due to differences in oncotic and hydrostatic pressure. This biological mass transfer is proceeded by their transit from the tissue interstitial space toward the lymphatics due to lower intraluminal concentration and pressure, thus resulting in lymphatic drainage.^[^ ^51^ ^]^ In vivo studies quantifying lymphatic drainage have implemented a similar methodology as the clinical imaging technique known as lymphoscintigraphy,^[^ ^52^ ^]^ where the clearance of an injected radiolabeled or fluorescent tracer from the interstitial space is monitored as it is collected by the lymphatics. Such measurements have been widely implemented for various in vivo systems (mouse, rat, rabbit) along different dermal sites and with tracers of different molecular weights, suggesting clearance rates in the range of ≈0.02–0.005 min^−1^.^[^ ^53^, ^54^, ^55^, ^56^, ^57^, ^58^ ^]^

To measure lymphatic clearance/drainage in our in vitro engineered lymphatics, we implemented an approach (Figure 3c), similar to that previously reported by Tien and colleagues.^[^ ^20^ ^]^ Briefly, a hydrostatic pressure difference (≈10 Pa) was established to drive interstitial flow through the gel matrix and toward the lymphatics at an average velocity of 1 µm s^−1^, corresponding to physiological flow conditions (Figure S1b, Supporting Information). Simultaneously, diluted fluorescent dextrans, with molecular weights representative of soluble interstitial macromolecules and proteins, are introduced into the higher‐pressure media channel. As the tracer is transported through the gel compartment and drained at the opposite media channel (lymphatics channel), we monitor the increase in fluorescence over time which is then used to calculate the corresponding solute drainage rate (Figure 3b,c). We utilized this assay to interrogate differences in lymphatic drainage for different solutes using four experimental conditions at the same time point: 1) a cell‐free gel, 2) a gel with a lymphatic endothelial cell monolayer on the left gel face, 3) a lymphatic network formed by growth factors alone, and 4) a lymphatic network formed by interstitial flow alone.

In the gel system, solute drainage rates fell within or below a value of 0.005 min^−1^ which barely recapitulates in vivo measured rates of solute drainage. For additional quantitative insight, we implemented a scaling analysis to characterize the relative importance between diffusive and convective transport using the Peclet number (*Pe*), a ratio of diffusive to convective time scales:

(1) $$Pe=\frac{u/L}{D/L^{2}}=\frac{uL}{D}$$

where *u* indicates the local velocity, *L* is the characteristic length of the system, and *D* denotes the diffusion coefficient of the solute. Considering the interstitial fluid velocity (1 µm s^−1^), width of the gel region, and varying diffusive coefficients between solutes, we find that *Pe* ranges from 8–26. Thus, in the gel system, transport is mostly dominated by convection.

In the lymphatic monolayer system, we observe a significant drop in solute drainage rate as the molecular weight of the tracer is increased beyond 10 kDa (*p‐value* = 0.03, 3 kDa vs 70 kDa). This trend is also observed in previous experiments studying blood microvascular systems,^[^ ^59^, ^60^ ^]^ in which both convective and diffusive transport rates decrease, with increasing tracer molecule size, as a result of the inherent difficulty of larger molecules to pass between the small dimensions of the endothelial intercellular space.^[^ ^55^ ^]^ In fact, while the interstitial fluid continues to travel at a physiological average velocity throughout the system, the introduction of the lymphatics as a monolayer in our microfluidic system locally imposed an additional barrier for large solute transport (>10 kDa) which effectively reduced drainage rates to sub‐physiological levels with larger molecules.

Next, we evaluated the drainage rates for the 3D lymphatics, engineered by lymphangiogenic induction with either growth factors or high interstitial flow (Figure 3d). For the full range of dextrans with varying molecular weight, our engineered lymphatic microvasculature achieved physiological levels of interstitial solute clearance, conversely to the other systems (bare gel and lymphatic monolayer). This can be explained by additional scaling arguments found below in Section 2.3.

We also examined the drainage rates for two biologically relevant proteins: avidin, extensively implemented for therapeutic particle conjugation,^[^ ^61^ ^]^ and albumin, the most ubiquitous plasma protein, responsible for maintaining oncotic pressure homeostasis in the bloodstream.^[^ ^62^ ^]^ The experimental measurements further validated that the bare gel or lymphatic monolayer system failed to recapitulate physiological drainage rates for these proteins (Figure 3e). However, systems that incorporate 3D lymphatic microvasculature recurrently exhibit protein drainage rates comparable to the in vivo‐measured values (Figure 3e). Slight differences in drainage rates between avidin and albumin could be attributed to electrostatic interactions between the negatively‐charged glycocalyx endothelial layer (Figure S5, Supporting Information) with the opposite charge nature of each protein (avidin positive, albumin negative).^[^ ^59^ ^]^

Our comparisons with in vivo data, indeed, highlight similarities in physiological drainage functionality between animal‐based models and our on‐chip lymphatics. However, it's crucial to interpret these measurements within the broader context of in vivo dynamics observed in both animals and humans. Recognizing its potential and limitations, this preliminary investigation aims to establish relevant metrics for lymphatic drainage functionality and establish a foundation for more specialized in vivo human studies.

### 2.3 Transport Phenomena Analysis of Solute Drainage in Engineered On‐Chip Lymphatics

**Figure 4:**
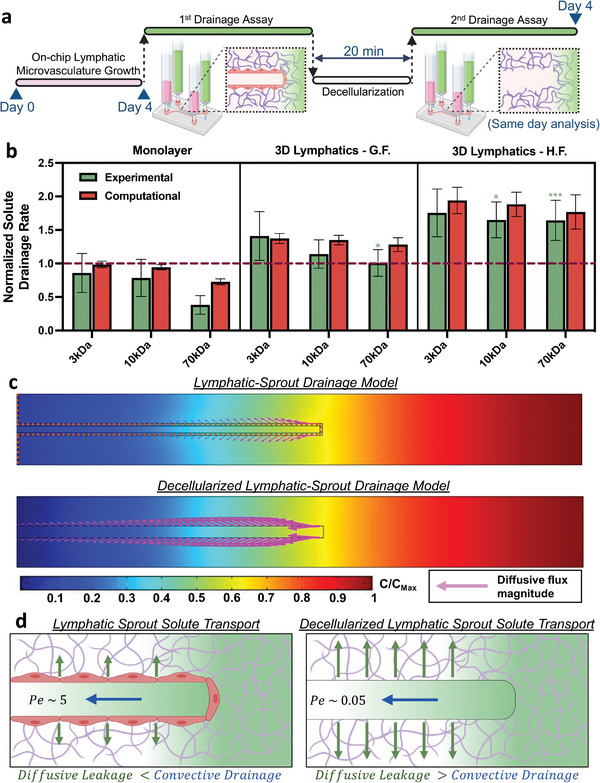
Drainage of interstitial molecules in on‐chip engineered lymphatics. a) Schematic of the experimental protocol to evaluate normalized drainage rates in our microfluidic systems. An initial solute drainage assay is performed similarly to Figure 3b. After decellularization of the system, a second drainage assay is run within the same device. This second (decellurized) drainage rate value is utilized to normalize the first drainage rate measurement. b) Normalized solute drainage rates (relative to their corresponding decellularized sample) for dextrans of varying molecular weight. Experimental data shown corresponds to the mean ± S.E.M., *n* = 2–4 samples (microfluidic devices) per condition, and *m* = 3 ROI images per sample. Statistical significance, between experiments, is reported with respect to the same molecular weight tracer in the monolayer system (color‐guided) in the gel‐only system, where *p < 0.05, **p < 0.01, ***p < 0.001 (based on the two‐tailed *t*‐test). Computational data shown corresponds to the mean ± S.E.M., *n* = 3‐4 conditions corresponding to iterations in the reflection coefficient‐parameter value. c) Axisymmetric 2D computational model for lymphatic and decellularized solute drainage, developed in COMSOL Multiphysics, depicting the spatial concentration of solutes (representative of 10 kDa dextran solution) at a time 300 s after the onset of flow from right to left in the transient model, also showing the magnitude of diffusive flux (length of magenta arrows) permeating outwards from the lumen. d) Schematic of solute transport models for lymphatic or decellularized sprouts where the relative competition between convective drainage and diffusive leakage are highlighted schematically and quantified by the Peclet number (*Pe*) for each system.

Beyond validating the physiological drainage functionality of our engineered lymphatics, we sought to elucidate the fundamental transport phenomena that gave rise to these distinctive drainage rates. In an effort to dissect differences in transport mechanisms contributed by the lymphatic endothelium across systems, an additional solute drainage rate assessment was conducted after decellularizing the on‐chip lymphatics samples (**Figure** **4**a). From this assessment, we experimentally evaluate if the lymphatic endothelium dampens or enhances the clearance of solutes in each system by normalizing the drainage rate of the initial lymphatic system with its corresponding decellularized measurement. Such results demonstrated that for the monolayer lymphatics, the addition of a lymphatic endothelium reduced drainage as represented by values falling below one when normalizing the original drainage rates by the corresponding decellularized measurements (Figure 4b. Conversely, for both 3D lymphatic systems, we observe values greater than one which translates to increased drainage due to the presence of the lymphatic endothelium within the 3D vascular structures. These data suggest that a 3D lymphatic endothelial vasculature contributes to the physiological drainage functionality, beyond just facilitating an increased vascular surface and additional microchannels for fluid and solute transport.

Given that our previous assessment is limited to a bulk measurement of interstitial solute drainage, we edified an alternate approach to characterize the spatial and temporal distribution of solutes during lymphatic collection and drainage. For this, we implemented an in silico model, developed using COMSOL Multiphysics, which recapitulated the solute drainage assay for an axisymmetric 2D geometry of a single lymphatic vessel within the gel region and its decellularized counterpart (Figure 4c). Both the geometry and experimentally measured transport properties were taken into account to develop these models (Table S1, Supporting Information). From these simulations, we identified two major transport events that determined the overall flux of interstitial solutes into the lymphatics. Initially, as solutes enter the lymphatic vessel, driven by fluid pressure and concentration differences, the intravascular fluid carries solutes at higher flow velocities due to significantly lower hydraulic resistance imposed by the lymphatic lumen, compared to the ECM‐gel region. This underpins the drainage capability of the lymphatic vascular system. We further validated this observation using scaling arguments (see Supporting Information section “*Scaling Analysis on Lymphatic Solute Drainage*”) by which we confirm that the hydraulic resistance of the lumen is ≈3 orders of magnitude less than the resistance of the gel region. Thus, driving the local drainage and collection of interstitial fluid and biomolecules into the distal lymphatic capillaries. Furthermore, scaling analysis with experimental values validates that the lymphatic endothelium imposes negligible hydraulic resistance, thus the convective flux of solutes into the lumen compartment between systems (lymphatic sprout and decellularized) is of similar magnitude.

Interestingly, as solutes travel faster within these channels a second phenomenon arises where significant concentration differences develop in the transverse direction of flow. This translates into a diffusive flux of molecules that exit the lumen (which we termed diffusive leakage) that decreases the effective amount of solutes drained by the lymphatics. While both the lymphatic and decellularized models facilitate the entry of interstitial molecules to similar degrees, the presence of a lymphatic endothelium reduces the diffusive leakage (Figure 4c), thereby increasing the drainage rates of all solutes, especially those of high molecular weight. Thus, providing a thin endothelial barrier that prevents the diffusive‐driven exit of intravascular solutes during drainage increases the drainage rate of the lymphatic system.

Although the implemented model captures the transport of just a single lymphatic sprout, we sought to translate these results to a comparable basis with the experiments. To evaluate the accuracy of our computational analysis, we monitored concentration variations over time at the outlet during drainage, which corresponds to the increase in fluorescence in our experimental system (Figure 3c). Subsequently, we calculated the ratio of drainage rates between the lymphatic sprout and the decellularized model, similar to the normalized measure from the experimental drainage assay (Figure 4a). By this comparison, we found a high degree of agreement between our experimental measurements and computational results, across the different systems and for solutes/tracers of varying molecular weight (Figure 4b); hence supporting our computational framework to describe the underlying transport phenomena and parameters that give rise to the distinctive drainage rates in the experimental system. We also performed scaling analysis to further support these findings by evaluating the Peclet number with appropriate adjustments to the scaling arguments (see Supporting Information section “*Scaling Analysis on Lymphatic Solute Drainage*”). We calculated that the Peclet numbers are in the range of 0.02 to 0.06, for the decellularized system, and 1.2 to 7, for the lymphatic sprout model, which implies that the presence of the lymphatic endothelium is sufficient to shift transport from diffusive to convective dominance when the endothelium is present. (Figure 4e). This consequently enhances solute drainage rates compared to bare, empty channels which is also consistent with the normalized drainage measurements presented. We view this analysis as an extension of the fundamental work by Thompson et al.,^[^ ^20^ ^]^ where they study the design principles that determine lymphatic drainage by pre‐pattering single vessel channels followed by lymphatic cell seeding; however, their experimental study was limited to a single lymphatic‐like capillary. In this work, we are able to confirm that this biological transport phenomenon also applies to a tissue‐scale engineered lymphatic network. Furthermore, these results provide theoretical insight into how we should rationally engineer 3D lymphatic tissue vasculature to recapitulate physiological drainage (see Supporting Information section “*Blood‐to‐Lymphatic Protein Transport Analysis”)*.

### 2.4 Immune Recruitment by Engineered Lymphatics is Coordinated by Inflammatory‐Induced Chemotactic Signals

**Figure 5:**
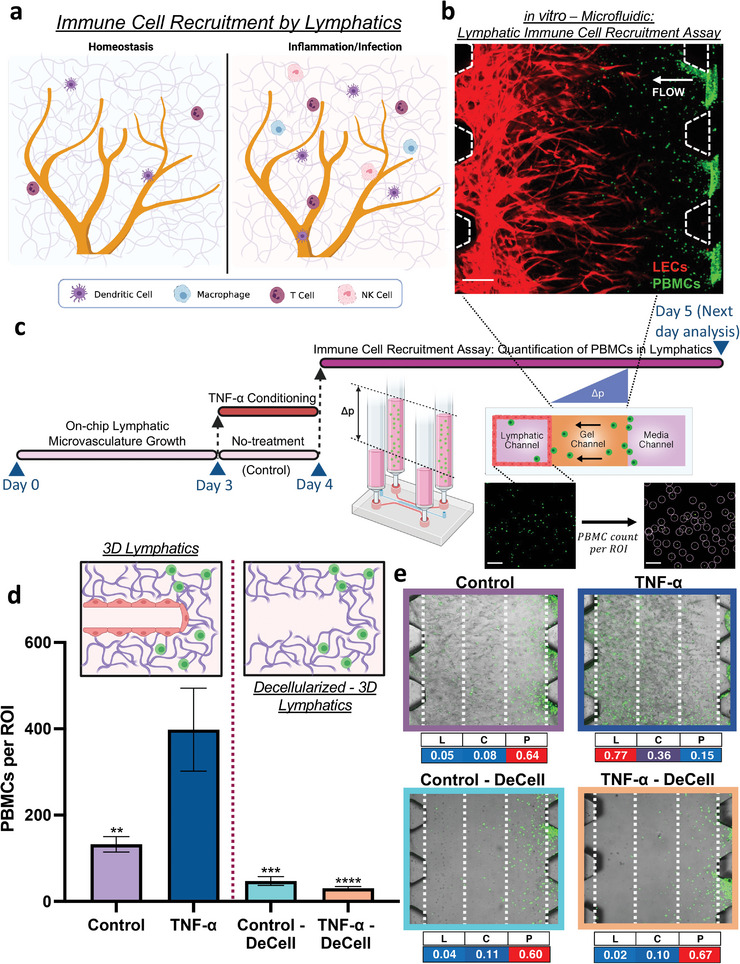
Modeling pathological immune cell recruitment with on‐chip engineered lymphatics. a) Schematic showing immune surveillance and recruitment in the lymphatic microenvironment according to the tissue state. b) Representative image of the PBMCs infiltration assay where the PBMCs (green) migrate through the ECM toward the lymphatics (red). Scale bar is 200 µm. c) Protocol and schematic of the experiments to evaluate immune cell recruitment in our microfluidic system where PBMCs are introduced into the adjacent media channel under a hydraulic pressure difference inducing pathological flow toward TNF‐α pre‐conditioned or untreated lymphatics. After 24 h, PBMCs that infiltrated the lymphatic gel region are counted per region of interest (ROI). d) Quantitative analysis of the PBMCs that reach the lymphatic region performed on high‐flow engineered lymphatics with certain devices stimulated with TNF‐α and/or decellularized. Data shown corresponds to the mean ± S.E.M., *n* = 3–4 samples (microfluidic devices) per condition, and *m* = 3 ROI images per sample. Statistical significance system significance is reported with respect to the TNF‐α stimulated lymphatics, where **p < 0.01, ***p < 0.001, ****p<0.0001 (based on the one‐way ANOVA test). e) Representative images and quantitative heat map analysis (below the image) correspond to PBMC distribution (green) within the gel region for each experimental condition. The table columns for L, C, and P correspond to the gel region with lymphatics, central gel region, and region adjacent to where the PBMCs are introduced, respectively.

In addition to regulating the transport of diluted proteins and other macromolecules in the interstitium, the lymphatic vasculature is essential for immune cell trafficking during host immune responses.^[^ ^63^ ^]^ In the event of tissue infection and inflammation, local stromal cells are activated by pathogenic signals, such as bacterial lipopolysaccharide, and respond with the release of inflammatory cytokines including tumor necrosis factor‐alpha (TNF‐α), transforming growth factor‐β (TGF‐β), interleukins, amongst others.^[^ ^64^ ^]^ Once the tissue site is primed by pro‐inflammatory signals, innate and adaptive immune cells are recruited by blood and lymphatic endothelial‐secreted chemokines, which then initiates immune activation and response^[^ ^24^, ^64^ ^]^ (**Figure** **5**a). Thus, in order to adequately study the signaling and migratory events that lead to the recruitment of immune cells to the lymphatics, the full range of fluid, protein, and cellular transport phenomena need to be recapitulated in in vitro models.

In addition to modeling lymphatic drainage of interstitial proteins, our platform is capable of recreating the pathological microenvironment that facilitates the intravascular recruitment of immune cells by the lymphatics. Recombinant‐human cytokines can be introduced into the system to mimic stromal‐secreted inflammatory factors. Additionally, immune cells can be introduced into the system at the adjacent media channel while establishing the appropriate hydraulic pressure difference across the gel compartment to impart a pathological level of interstitial flow toward the lymphatic channel (Figure 5b,c). As a model for immune cell response, we isolated human peripheral blood mononuclear cells (PBMCs) which include a broad population of immune cells that are naturally found in the systemic circulation, and are known to extravasate and migrate into the lymphatic periphery. We can quantify the magnitude of lymphatic‐elicited immune response by counting the number of PBMCs recruited to the lymphatic channel after 24 hours of induction (Figure 5c). In initial experiments, PBMCs consistently migrated into the opposite channel at higher numbers for devices incorporating lymphatics (high‐flow stimulated, growth factor‐grown, and monolayer), in comparison to the bare gel system (Figure S8, Supporting Information). Given such similar trends for lymphatic‐based systems, we focused the rest of our studies on solely high interstitial flow‐engineered lymphatics.

To validate the increased lymphatic recruitment of immune cells under inflammatory conditions, devices with high flow‐engineered lymphatics were treated with TNF‐α prior to PBMC perfusion, in parallel with a set of devices without TNF‐α exposure serving as the control. Based on our solute drainage measurements, we also considered decellularized samples to determine if the increased infiltration is solely due to changes in the physical structure of the matrix, by the invading lymphatic sprouts, which could then facilitate the migration of the immune cells. Results from this set of experiments validated that, under inflammatory stimulus (TNF‐α pre‐treatment), lymphatics significantly increase the recruitment of PBMCs (Figure 5d). Additionally, decellularized samples consistently exhibited lower infiltration numbers regardless of whether the devices were preconditioned or not with TNF‐α. To further examine the preferential migration and infiltration of PBMCs, confocal imaging of the gel region was done to visualize the spatial distribution of PBMCs immediately after performing the infiltration assay. Both images and quantitative data indicate that PBMCs where preferentially localized closer to the media region where they are introduced, for all conditions except the TNF‐α‐stimulated lymphatics (Figure 5e). For the latter, the highest population of PBMCs corresponded to the region co‐localized with lymphatic vessels. These data suggest that, under inflammatory stimulus, our on‐chip lymphatics provide directional cues that elicit the preferential migration and infiltration of PBMCs.

**Figure 6:**
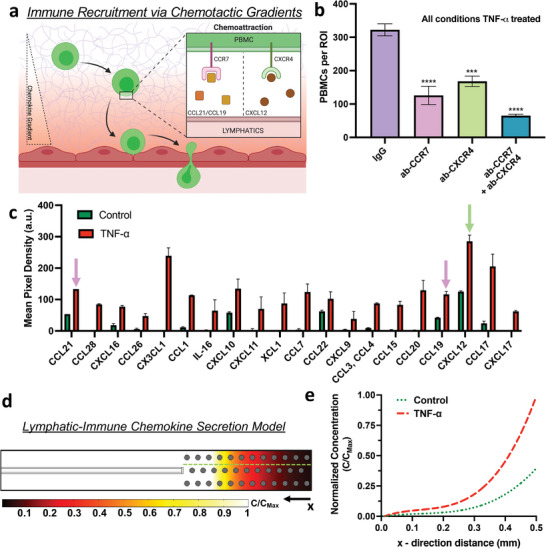
Flow‐induced concentration gradient of chemotactic factors facilitates immune recruitment by on‐chip lymphatics. a) Schematic diagram of the chemotactic signaling axes that recruit immune cells via concentration gradients of the corresponding chemokines during an inflammatory response. b) Quantitative analysis from the PBMCs infiltration assay performed on high‐flow engineered lymphatics stimulated with TNF‐α and with corresponding conditions of PBMCs reconstituted in neutralizing antibodies or IgG isotypes. Data shown corresponds to the mean ± S.E.M., *n* = 3‐4 samples (microfluidic devices) per condition, and m = 3 ROI images per sample. Statistical significance is reported with respect to the experimental condition with PBMCs reconstituted in IgG isotypes, where ***p < 0.001, ****p < 0.0001 (based on the one‐way ANOVA test). c) Quantitative analysis of cytokine secretion from supernatant collected from lymphatics grown under interstitial flow with one experimental group pre‐conditioned with TNF‐α. Data shown corresponds to the mean ± S.E.M., *n* = 2 samples (cytokine detection spots). Arrows indicate the corresponding immune cell receptors in accordance with the color from the previous graph. d) Computational model results for lymphatic chemokine transport, developed in COMSOL Multiphysics, illustrates the steady‐state normalized concentration of chemokines. Grey‐filled circles represent PBMCs as impermeable objects in the gel domain. Green, dashed line indicates the line probe from which the concentration plot e) is generated showing differences in secretion rate by untreated (control) lymphatics and TNF‐α‐stimulated samples.

Immune cell recruitment to specific sites is guided by chemokines.^[^ ^65^ ^]^ These chemotactic cytokines provide coordinated delivery of cell‐secreted ligands to specific immune cell surface receptors^[^ ^66^ ^]^ (**Figure** **6**a). Among the most studied immune chemotactic pathways involve the lymphatic secreted ligands CCL21 and CCL19 that attract immune cells via their CCR7 receptor in various pathological conditions including immunogenic response and cancer metastasis,^[^ ^67^, ^68^, ^69^ ^]^ and stromal/endothelial secreted CXCL12 that coordinates the homing of immune cells for immune maintenance and development by their CXCR4 surface receptor.^[^ ^70^, ^71^ ^]^ To determine if these receptors are elicited during inflammatory immune recruitment in our tissue‐engineered system, we performed receptor‐blocking experiments by incubating PBMCs in a buffer solution (containing CCR7 and/or CXCR4 antibodies, or control isotypes), prior to performing the infiltration assay with HF‐grown lymphatics stimulated with TNF‐α. For this panel of experiments, we observed a consistent and significant decrease in the number of infiltrated PBMCs when their cell surface receptors, CCR7 and/or CXCR4, were functionally blocked, compared to the IgG isotype control, despite being introduced into devices with inflammatory‐stimulated lymphatics (Figure 6b). Furthermore, analysis of the spatial distribution of PBMCs within the gel region revealed that solely for the IgG isotype control samples, the PBMCs preferentially migrated and co‐localized at the inflamed‐lymphatics region (Figure S9, Supporting Information). All other samples with neutralizing antibody treatment displayed higher PBMC numbers closer to the media channel. Thus, the CCR7 and CXCR4 immune cell surface receptors, responsible for immune homing, are elicited in our engineered pathological model for immune cell recruitment by the lymphatics in response to local inflammatory stimulus. However, we anticipate that additional immune cell receptors are co‐activated during the recruitment of PBMCs during infiltration. As such, future studies to interrogate activated immune chemotactic receptors during lymphatic recruitment can be performed with our platform.

To comprehensively validate the increased activation and recruitment of PBMCs by lymphatic‐secreted chemokines during inflammation, we quantified cytokine expression by collecting the supernatant from lymphatic cells cultured under similar experimental conditions. Quantitative analysis of the cytokine concentrations for both experimental conditions revealed a consistent increase in chemokine secretion by the TNF‐α‐conditioned lymphatics, with an ≈2.5‐fold increase for CCL19, CCL21, and CXCL12 (Figure 6c). We conclude therefore that the immune surface receptors CCR7 and CXCR4 mediate the increased infiltration of PBMCs, while lymphatics secrete corresponding ligands that activate these chemotactic receptors within our inflammatory immune recruitment model.

When considering the physio‐/pathological environment by which these chemokines are secreted into the extracellular space, most of them remain soluble as passive transport drives their spatial distribution from a localized source toward lower concentration regions.^[^ ^72^, ^73^, ^74^ ^]^ Thus, facilitating graded chemical concentrations that provide homing and directional cues for the directed migration and recruitment of the immune cells to a specific tissue or vasculature.^[^ ^75^, ^76^ ^]^ But we wondered if the interstitial flow generated under pathological conditions might impede chemokine diffusion in the opposite direction, thereby preventing the chemokine gradient from influencing the PBMCs. We, therefore, sought to demonstrate that these chemotactic conditions could be established and maintained in the presence of high interstitial flow toward the lymphatics. To address this question, we turned to our computational lymphatic drainage model, incorporating the migrating PBMCs as solid (impermeable) spheres embedded in the gel region. Based on a quasi‐steady state approximation (supported by scaling arguments, Supporting Information), we computed the concentration gradients of lymphatic‐secreted chemokines (Figure 6d). The numerical results indicate that the spatial distribution of the chemokine, although skewed by the high interstitial flow speeds (consistent with a Peclet number ≈ 10),^[^ ^77^ ^]^ follows a concentration gradient leading toward the lymphatics (Figure 6e). From the cytokine array analysis, we found that the chemokines of interest are secreted at a 2.5x increased rate for inflammatory‐stimulated lymphatics. We applied this relative increase to the flux of species by the lymphatic sprout in our model to which we found a significantly steeper profile in the concentration gradient of the chemokines (Figure 6e). As extensively studied in vitro,^[^ ^78^, ^79^, ^80^ ^]^ such an increase in spatial gradients of chemotactic factors results in faster and more persistent migration patterns by immune cells, which is in line with our experimental findings that higher numbers of immune cells infiltrate our inflamed lymphatics model.

## 3 Conclusion

In this work, we address the need for a physiologically relevant in vitro model that recapitulates critical aspects of the in vivo lymphatics, including their tissue architecture and physiopathological functionality. For this purpose, we implemented a microfluidic‐based cell culture system that allowed us to compartmentally culture lymphatic endothelial cells under the appropriate biological stimuli to induce their natural self‐organization into in vivo‐like capillaries. Furthermore, we leveraged the capabilities of our microfluidic system to recreate the biological transport of fluid, interstitial solutes, and immune cells which allowed us to perform functional assays with the on‐chip engineered lymphatics. On the basis of these studies, we successfully recapitulated lymphatic in vivo functions pertaining to their physiological solute drainage and immune‐lymphatic pathological response. Such results support the use of our microphysiological platform for pre‐clinical applications that would be technically challenging to perform with in vivo models, such as characterizing lymphatic absorption of subcutaneously delivered therapeutic antibodies and screening immunogenic response of genetically‐modified immune cells that transit across the lymphatic interface to reach their corresponding cellular target. Building upon our novel techniques, we anticipate future studies that will further narrow the technological gap between in vitro models and human‐based in vivo conditions, thereby establishing the foundation for the clinical translation of such microphysiological systems.

## 4 Experimental Section

### Microfluidic Device Design and Preparation

The microfluidic device was based on earlier designs from the lab^[^ ^81^, ^82^, ^83^ ^]^ with three, parallel fluid channels. The middle channel was lined by a series of trapezoidal posts at the edges adjacent to the other fluid channels. These provide adequate surface tension to facilitate the compartmentalization of the injected extracellular matrix in the middle region. The side channels were then utilized as medium channels that allow the exchange of nutrients and metabolic waste, as well as to supply growth factors at specific boundaries of the gel region. Additionally, the length scales of the system were optimized to facilitate lymphatic vascularization in the middle, gel channel. The distance between media channels, which defines the width of the gel region, is 1.2 mm – sufficiently short to allow the steady diffusion of growth factors from one media channel to the other within several hours, while providing sufficient distance for the lymphatic cells to generate vascular sprouts in the gel region.^[^ ^81^ ^]^ On a similar basis, a width of 1 mm was set for the media channels. The device height, 300 µm, was chosen to maximize the 3D space of lymphatic vascularization, while still facilitating high‐resolution confocal imaging. Finally, the length of the media‐gel interface was extended to 1 cm to approach a tissue‐relevant scale while still maintaining a small device footprint.

Microfluidic devices were fabricated by soft lithography from SU‐8 coated silicon molds similarly to previous protocols.^[^ ^84^ ^]^ Briefly, molds were prepared by photopolymerizing a 300 µm thick SU‐8 photoresist (Micro‐Chem, USA) on the silicon wafer. After developing the SU‐8 layer, the wafer was silanized overnight in a vacuum desiccator to facilitate the passivation of the surfaces, thus preventing PDMS adhesion during removal. A 10:1 mix of PDMS (Sylgard 184, Ellsworth Adhesives, USA) and curing agent was then poured onto the mold, allowed to degas in a desiccator for ≈30 min, and polymerized at 70 °C for at least 2 hrs. PDMS was then removed from the mold and cut into individual devices. Scotch tape was used to further clean the surface of the device removing dust and particulates. To allow upper access to the fluid channels, ports were punched using a 1.2 mm biopsy punch for the gel channel, and a 6 mm or 4 mm biopsy punch for the media channels in devices used to grow the lymphatic microvasculature via growth factors or interstitial flow, respectively. After dry sterilization of the devices, the surface was treated with plasma (Harrick Plasma, USA) for 90 s, and then bonded to a coverslip slide. After plasma bonding, devices were left overnight to recover hydrophobicity and kept sterile until use.

### Cell Culture

Human dermal lymphatic microvascular endothelial cells (CC‐2543, HDLMEC, Lonza, USA) were cultured in Vasculife Endothelial Medium (Lifeline, LL‐0003) supplemented with 6% FBS (Invitrogen). HDLMEC were transduced to express cytoplasmic RFP using LentiBrite RFP Control Lentiviral Biosensor (EMD Millipore, 17–10409) as described by the vendor. Cells were cultured at 37 °C and 5% CO_2_ in a humidified incubator with media replacement every second day. HDLMEC was used in experiments before reaching confluence, between passages 6–8.

### Growth Factors, Fluorescent Tracers, and Antibodies

All reagents were reconstituted to a stock solution as recommended by the corresponding vendor, and then diluted to desired concentrations. Vascular endothelial growth factor‐c (VEGF‐C, R&D Systems), angiopoietin‐1 (ANG‐1, R&D Systems) and hepatocyte growth factor (HGF, Peprotech) were all used at a 100 ng mL^−1^ dilution in the cell culture medium for specified experimental conditions. Immunostaining of the lymphatic vasculature was performed using PE‐conjugated anti‐human podoplanin (Biolegend), Alexa Fluor 647‐conjugated anti‐human lymphatic vessel endothelial receptor‐1 (LYVE‐1,R&D Systems) or Alexa Fluor 488‐conjugated anti‐human laminin (R&D Systems) to image the cell surface and DAPI (Invitrogen) or Alexa Fluor 594‐conjugated anti‐human Prox1 (Biolegend) to image cell nuclei at a 1:100 and 1:1000 dilution from stock in washing buffer (0.5% BSA/DPBS), respectively. For diffusive and convective transport measurements, fluorescent dextran: 3 kDa‐Cascade Blue (Thermofisher), 10 kDa‐Cascade Blue (Thermofisher), or 70 kDa‐Fluorescein (FITC, Thermofisher) was supplemented in the cell culture medium at concentration of 100 µg mL^−1^. In a similar set of experiments, Fluorescein‐conjugated avidin (Thermofisher) or Alexa Fluor 647‐conjugated albumin from bovine serum (Thermofisher) was introduced into the system at a concentration of 100 µg mL^−1^. For neutralizing antibody experiments, PBMCs were incubated in a buffer solution with their respective antibody blocker at a concentration of 5 µg mL^−1^: human CCR7 antibody (MAB197‐SP, R&D), human CXCR4 antibody (MAB172‐SP, R&D), and a combine human IgG_2A_ and IgG_2B_ isotype control (MAB003 and MAB004, R&D).

### Device Seeding and Microvascular Culture

Fibrinogen from bovine plasma (Sigma) was dissolved for at least 3 h at 37 °C in Dulbecco's Phosphate‐Buffered Saline (DPBS, Lonza) at 5 mg mL^−1^, twice the final concentration. A thrombin (Sigma) stock solution was made at 100 U mL^−1^ in 0.1% w/v bovine serum albumin (BSA) solution and stored at −70 °C. Thrombin was diluted in Vasculife Endothelial Basal Medium to a concentration of 4 U mL^−1^. Solutions were mixed via pipetting, over ice, in a tissue culture hood at a 1:1 ratio to produce a fibrin solution with a final fibrinogen concentration of 2.5 mg mL^−1^. The mixture was then pipetted into the device using the gel filling ports. Devices were placed in a humidified enclosure and allowed to polymerize at room temperature for 15 min.

Human Plasma Fibronectin (EMD Millipore) was diluted to a concentration of 100 µg mL^−1^ in DPBS, prior to being injected into one of the media channels where the HDLMEC would be seeded in order to facilitate their adhesion to the walls of the device. While the devices were left incubating with the fibronectin solution for at least 30 min, HDLMEC were trypsinized (Lonza, USA) and resuspended to a concentration of 3 × 10^6^ cells mL^−1^. After incubation, fresh media was introduced into the fibronectin‐coated channels and aspirated, followed by perfusion of 30 µL of the cell suspension into the channel. Immediately after cell seeding, devices were tilted by ≈120° and incubated for 15 min to facilitate the adhesion of cells on the gel‐media interface. Subsequently, devices were returned to their original position and fresh media was supplemented into the remaining media channel devoid of cells. A pressure difference of ≈10 Pa (1 mmH_2_O) was established between media channels with flow directed from the lymphatic media channel toward the opposite media channel to further assist the accumulation of cells at the interface. After 24 h of culture under these conditions, a confluent monolayer of lymphatic endothelial cells forms at the gel media interface, and the remaining unattached cells are aspirated.

Following the formation of a confluent lymphatic monolayer (Figure 2a), ≈350 µL plain Vasculife Endothelial Medium was replenished in the lymphatic media channel. To stimulate lymphatic sprout formation into the 3D gel region, ≈350 µL of cell culture medium supplemented with the specified growth factor was added to the adjacent media channel and replenished on a daily basis for up to 6 days. Previous dose‐response experiments were also performed (data not shown) for the concentration of growth factors, from which we identify a concentration threshold (100 ng mL^−1^), above which lymphatic sprouting is not significantly improved during the course of the experiments. For experiments where interstitial flow was implemented to stimulate lymphatic sprouting, a pressure head difference was applied using disposable 10 mL luer‐slip syringes (Kinesis) cut at the 4 mL label, then dry sterilized. Once the lymphatic monolayer was generated, syringes were gently press‐fit into the media ports and media was added to the reservoirs accordingly to the desired pressure difference (either 100 or 10 Pa for high flow (HF) and low flow (LF), respectively) and interstitial flow velocity (Figure S1b, Supporting Information). This simple reservoir setup was critical for our flow experiments. Due to its larger cross‐sectional area (1 cm diameter), it accommodates large volumes of media which in turn provides higher precision for establishing pressure differences across the gel while maintaining the hydraulic pressure head for long periods, with minimal pressure loss over a 24‐hr period (<10%). As such, the pressure difference only had to be re‐established on a daily basis

### Immunofluorescence Staining, Imaging, and Quantification

Growth of the lymphatic vasculature was measured every other day over a course of 6 days by taking epifluorescence images of their RFP signal on a Nikon Eclipse Ti‐S (Nikon Instruments, USA) at 4x with a numerical aperture of 0.13. These parameters permit an imaging thickness of ≈25 µm,^[^ ^85^ ^]^ which is similar to the thickness of histological sections used to quantify in vivo lymphatic morphology.^[^ ^86^ ^]^ The Lymphatic Vessel Analysis Protocol‐plugin in ImageJ (NIH) was utilized to measure morphological properties under the same protocol as implemented for lymphatic capillaries from tissue cryosections.^[^ ^87^ ^]^ The area of coverage was quantified from binarized images that showed the relative area within the gel invaded by the lymphatics. Vessel diameter was measured at 2 to 3 locations along each sprout, for a total of 5–20 sprouts per imaged area (depending on the number of available sprouts to measure accordingly to experimental condition). For immunofluorescence imaging, cells were fixed with 4% paraformaldehyde (Electron Microscopy Sciences) for 15 min, followed by permeabilization with 0.01% Triton X‐100 (Sigma) for 10 min. Subsequently, blocking was performed with 5% BSA (Sigma) and 3% goat serum (Sigma) for 1 hr at room temperature. All the reagents were diluted in DPBS. Cells were then incubated overnight at 4 °C with a corresponding protein‐antibody of interest. After incubation, the samples were washed 5x with washing buffer and stored at 4 °C. Confocal images were acquired with IX81 microscope (Olympus) equipped with Fluoview FV1000 Software (Olympus).

### Diffusion, Interstitial Flow, and Hydraulic Permeability Assessment

For characterization of the intrinsic transport properties of the gel, device gel seeding was followed as specified in the previous section but without the addition of cells and left to incubate overnight. Cell culture media supplemented with 70 kDa‐FITC dextran was added to one of the media channels and the middle, gel region was imaged at specified time intervals on a confocal microscope (Olympus FV‐1000) with custom enclosure for temperature and atmosphere control. Fluorescent signal obtained from the images depicted changes in the concentration profile over time due to diffusive transport of the molecules across the gel. Images were taken at the midplane with a 10x objective, then analyzed using ImageJ (NIH) to extract the fluorescence intensity profile across the width of the gel channel. Computational simulations were performed to obtain the diffusion coefficient of the tracer within the gel region (Supporting Information).

To quantify interstitial fluid velocity corresponding to the established pressure difference across the gel, a variation of the Fluorescence Recovery After Photobleaching (FRAP) technique was implemented as previously reported.^[^ ^88^ ^]^ Devices were perfused with 70 kDa‐FITC dextran‐supplemented media and left overnight to ensure its uniform distribution throughout the gel. Subsequently, 3 to 5 small regions of interest (ROIs) 30 µm in diameter were photobleached by setting the confocal laser power to 100% for ≈5 s within the gel matrix. Time‐lapse images were captured at 10x immediately after photobleaching every 1.5 s. By measuring the translation of the photobleached spot centroid using the Matlab frap_analysis plugin,^[^ ^89^ ^]^ we extracted the interstitial fluid velocity at each pressure head difference. This assumes that: (1) the tracer molecule is small compared to the pore size of the fibrin matrix,^[^ ^90^ ^]^ and (2) the flow field is uniform, which is also valid since the length scale of the gel compartment is orders of magnitude greater than the distance over which the velocity varies close to the device wall (*w, h* >> *K* ^0.5^); thus, the flow velocity is nearly uniform across the gel, falling to zero over the small distance *K* ^0.5^ at the device walls.^[^ ^91^ ^]^ This measurement was repeated as the pressure difference was increased to obtain an experimental trend between the pressure offset (*Δp*) and interstitial flow velocity (*v*) to confirm the linear relationship (Darcy's Law – Equation 2), and determine the hydraulic permeability of the gel (*K*) given by:

(2) $$K\;=\frac{w\;\mu \;v}{\Delta p}\;$$

where *w* indicates the length over which the pressure drop is imposed (gel channel width), and *µ* corresponds to the fluid viscosity taken as 0.78 cP from previous studies.^[^ ^92^ ^]^

### Lymphatic Solute Drainage Rate Assessment

A solute drainage assay was implemented based on previous in vivo techniques and adapted for in vitro models.^[^ ^20^, ^93^ ^]^ On day 4 post‐seeding/culture, media supplemented with a fluorescent tracer was introduced into one of the media channels to establish a hydraulic pressure difference between media channels and drive flow through the gel at an average interstitial fluid velocity of ≈1 µm s^−1^, thus recapitulating physiological flows toward the lymphatics. Once the fluorescence intensity in the downstream media channel begins to rise, following a period of time (typically ≈10 min) allowing the tracers to reach the lymphatic network, a series of 4 ROIs at the media channel (initially solute‐free) are imaged as a confocal stack throughout the full height of the device (4 slices at 80 µm) at 10x every 2–4 min for up to 12 min to determine average fluorescent intensity over time. Additional images were acquired for the source channel, where the fluorescently‐conjugated solutes are originally introduced. The average fluorescence intensity of each corresponding ROI was extracted using ImageJ‐based quantification and used to calculate the solute drainage rate:

(3) $$Solute\;Drainage\;Rate\;=\;\frac{\Delta I_{v}}{\Delta t}\frac{1}{I_{s}}$$

where *ΔI_v_* indicates the increase in the average fluorescence intensity within the lymphatic vasculature over a time interval (*Δt*), and *I_s_* is the average intensity in the source channel. A fundamental assumption imposed by this metric is the linear increase in fluorescence intensity as the tracer drains into the lymphatic channel. Measurements from this assay by Tien and colleagues^[^ ^20^ ^]^ and our study (Figure S4, Supporting Information) validate a nearly linear trend in the fluorescence signal, corresponding to convection‐dominated transport of solutes into the lymphatic channel. However, we are measuring under transient non‐equilibrium conditions, thus fluctuations in the solute drainage rate can still arise throughout the duration of the assay. To validate the accuracy of our estimate, we implemented our computational model (see Supporting Information Section) to monitor the increase in concentration in the outlet until reaching saturation. Simulation results confirm that by just calculating the average solute drainage rate from the initial 10 min right after the tracer reaches the lymphatic channel, we are able to approximate the mean value from the overall variations in drainage rate (Figure S7, Supporting Information).

For devices containing lymphatic endothelial cells, an additional solute drainage rate assessment was conducted after decellularizing the system. This was done by washing away the cells with a detergent solution of 1% Triton X‐100 in DPBS for 10 min immediately after the first solute drainage measurement, followed by washing the previous fluorescent tracer using DPBS for 5 min. All the washing steps were done under a slight pressure head difference (≈10 Pa). Solute drainage rate measurements were repeated with decellularized devices to determine the relative difference between measured values prior to and post‐lymphatic decellularization. From supplemental experiments, no significant differences are observed in the transport properties of the ECM after decellularization with a solution of 1% Triton X‐100 (Figure S10, Supporting Information).

### Lymphatic Diffusive Permeability and Hydraulic Conductivity Assessment

To quantify diffusive transport across the lymphatic endothelium, a diffusive permeability assay was implemented based on previous protocols.^[^ ^59^ ^]^ Briefly, devices were carefully perfused (avoiding pressure imbalances that could skew diffusion across the gel) with a fluorescent tracer at the lymphatic media channel, and allowed to diffuse from the main channel to the lymphatic vessels, and finally through the endothelium. During the latter, confocal z‐stacks of >100 µm thick were collected in steps of 5 µm and at time intervals of 2 min. Considering the principle of mass conservation (Equation 4), the diffusive flux (*N_d_*) of the solutes from the lymphatic endothelium into the gel region can be quantified by taking the temporal derivative of the total concentration (*C*) in the gel control volume (*V_g_*):

(4) $$N_{d}=\frac{\partial }{\partial t}\int_{V_{g}}CdV=A_{s}P_{e}\Delta C$$

Since the total diffusive flux is driven by the concentration difference (*ΔC*) across the surface area of the vasculature (*A_s_*), and assuming that the fluorescence intensity is linearly proportional to the concentration of the fluorescent tracer, the diffusive permeability of the endothelium (*P_e_*) can be computed from:^[^ ^94^ ^]^

(5) $$P_{e}=\;\frac{\Delta I_{g}}{\Delta t}\frac{1}{I_{v,i}-I_{g,i}}\frac{V_{g}}{A_{s}}$$

where *ΔI_g_* is the increase of average fluorescence intensity in the gel region after a given time interval (*Δt*), respectively, and *I_v,i_* and *I_g,i_* indicate the initial, average fluorescence intensity within the lymphatic vasculature and gel volume, respectively. This measurement was repeated at 3 locations per device for fluorescent tracers of varying molecular weight.

Fluid transport across the lymphatics was characterized in terms of endothelial hydraulic conductivity. For this assessment, interstitial flow velocities (*v*) in devices with lymphatics were measured at the same pressure head differences (*Δp_total_*) used for the estimation of the hydraulic permeability. Following these measurements, and recognizing that the major sources of hydraulic resistance (ratio of differential pressure and volumetric flow rate) in the system originate from the gel and lymphatic endothelium,^[^ ^42^ ^]^ the mean differential pressure across the endothelium (*Δp_ec_*) can be estimated by:

(6) $$\Delta p_{ec}=\;\Delta p_{total}-\;\frac{w^{*}\;\mu \;v}{K}$$

where the differential pressure across the gel is governed by Darcy's Law, as previously described. A corrected average gel width (w*) is implemented here to account for the reduced gel area due to endothelial invasion, in cases where we have 3D lymphatic sprouts. This was done by performing confocal imaging to measure the volume and surface area contributed by the lymphatics in the gel region. Subsequently, by estimating the mean pressure drop contributed by the endothelium, the Starling equation could be applied to establish the proportionality between the pressure difference and fluid flux across the lymphatics, known as the hydraulic conductivity (*L_p_*), by which the previous assumptions we estimate an effective hydraulic conductivity:

(7) $$L_{p}=\frac{v}{\Delta p_{ec}}\;$$

where the oncotic pressure contribution is neglected assuming nearly homogenous concentration of solutes throughout the system. This quantification was performed for each configuration of the microfluidic system (monolayer, growth factors‐ and interstitial flow‐grown lymphatics). All transport parameters were measured and utilized for the simulations are listed in Table S1 (Supporting Information).

### Immune Cell Isolation and Recruitment Assessment

Human blood collected from healthy donors (Research Blood Components, Massachusetts, USA) with anticoagulant sodium citrate, was mixed at a ratio of 1:1 with a buffer solution, consisting of DPBS supplemented with 0.1% BSA (Sigma) and 1 mm of ethylenediaminetetraacetic acid (ThermoFisher), and carefully layered over Ficoll Paque Plus (Sigma) in a Corning 50 mL centrifuge tube. Then, the layered blood samples were centrifuged at 400 rcf for 35 min. Following centrifugation, the upper layer, consisting of plasma, was removed by aspiration, and the remaining buffy layer (containing PBMCs) was transferred to a separate centrifuge tube. Additional purification from any remaining platelets was performed by diluting the isolated PBMCs in buffer solution supplemented to the remaining volume of the 50 mL tube, and centrifuging the resuspended cells at 250 rcf for 10 min. Platelets mostly remained in the supernatant, which was aspirated, and PBMCs remained agglomerated at the bottom. This final step was repeated twice, as recommended by the standard isolation protocol to yield >90% of PBMC purity. All isolation procedures were done at room temperature. Immediately after purification, PBMCs were stained with Cell Tracker Green CMFDA Dye (Invitrogen) at a 10 µm concentration for 10 min, followed by washing with the buffer solution, and resuspended in cell culture media to a final concentration 1 × 10^6^ cells mL^−1^. For receptor blocking experiments, an additional incubation step was implemented before the final resuspension step, with PBMCs incubated in the buffer solution containing CCR7 and/or CXCR4 antibodies, control IgG antibodies or no treatment for 30 min. After the final resuspension, 200 µL of the PBMC solution (≈200 000 PBMCs) was perfused into the media channel devoid of lymphatic cells in devices at day 4 of cell culture. For some cases, devices were pre‐treated with 20 ng mL^−1^ of TNF‐α (Peprotech) overnight. Prior to PBMC perfusion, any remaining TNF‐α was washed away to ensure that inflammatory stimulus was solely applied to the lymphatics. Once the PBMCs were introduced into the devices, a hydraulic pressure head was established to drive flow toward the lymphatic channel at pathological interstitial fluid velocities (≈4 µm s^−1^), as estimated in inflamed tissues.^[^ ^31^, ^32^ ^]^ Following 24 hrs of this assay, epifluorescence images were acquired at the lymphatic channel, and the total number of PBMCs was quantified using the TrackMate‐plugin in ImageJ (Figure 5c). Additional imaging was done with fixed devices which included confocal imaging of the gel region to visualize the spatial distribution of PBMCs. Quantitative analysis of the images was performed by subdividing the gel into three equally‐spaced regions along its width (Figure 5e) and measuring the average fluorescence intensity of each region which correlated with the density of immune cells.

### Chemokine Secretion Analysis

Chemokine expression was quantified by collecting the supernatant from lymphatic cells cultured under experimental conditions similar to those for the immune recruitment assay. Prior to collection, lymphatic endothelial cells (≈10^6^ cells) were seeded on a Corning Transwell Membrane Inserts with 0.4 µm pores (Sigma). After 24 hrs of culture, a lymphatic monolayer was established on the transwell insert, and 1 mL of fibrin gel was injected on top of the monolayer and allowed to polymerize. Subsequently, cell culture media was added to the top of the gel inducing interstitial flow (≈4 µm s^−1^) toward the lymphatics with one of the transwell having cell culture media supplemented with 20 ng mL^−1^ of TNF‐α. Following 24 hrs of culture under these conditions, the supernatants were collected and assayed in a Proteome Profiler Human Chemokine Array (ARY017, R&D systems) following manufacturer's protocol. Chemiluminescent imaging was performed using Alpha Innotech (USA), and the generated images were analyzed using the Protein Array Analyzer‐plugin in ImageJ.

### Statistical Analysis

Statistical analysis was done in Graphpad Prism (Graphpad, USA). All data shown represent experiments with *n* = 2–4 individual devices per condition. Reported values correspond to averages over these devices with error bars representing the standard error of the mean. A two‐tailed t‐test was used when comparing the difference between two groups, while comparisons for multiple groups were analyzed by ANOVA followed by a Tukey post‐hoc test. All tests resulting in a *p‐value* less than 0.05 were considered statistically significant and were grouped as *p < 0.05; **p < 0.01; ***p < 0.001; ****p < 0.0001; ns (not significant), *p* > 0.05.

## Conflict of Interest

R.D.K. is a cofounder of AIM Biotech which markets microfluidic systems for 3D cell culture. R.D.K. also receives research support from Amgen, Roche, Boehringer Ingelheim, Glaxo‐Smith‐Kline, Novartis, Takeda, Eisai, Visterra Merck EMD Serono and AbbVie.

## Supporting information

Supporting Information

## Data Availability

The data that support the findings of this study are available from the corresponding author upon reasonable request.
