# Supplementary material for: Microfluidic‐Based Reconstitution of Functional Lymphatic Microvasculature: Elucidating the Role of Lymphatics in Health and Disease
Source: Adv Sci (Weinh). 2023 Dec 7;11(5):2302903. doi: 10.1002/advs.202302903 (PMC10837354; doi:10.1002/advs.202302903)
Supplement: Supplementary file 1 — Supporting Information [file ADVS-11-2302903-s001.pdf]

ADVANCED SCIENCE
Open Access

Supporting Information

for *Adv. Sci.*, DOI 10.1002/advs.202302903

Microfluidic-Based Reconstitution of Functional Lymphatic Microvasculature: Elucidating the Role of Lymphatics in Health and Disease

*Jean C. Serrano, Mark R. Gillrie, Ran Li, Sarah H. Ishamuddin, Emad Moeendarbary and Roger D. Kamm**

Supplementary Information

Microfluidic-based reconstitution of functional lymphatic microvasculature: Elucidating the role of lymphatics in health and disease

*Jean C. Serrano, Mark R. Gillrie, Ran Li, Sarah H. Ishamuddin, Emad Moeendarbary, Roger D. Kamm**

Supplementary Text

Finite Element Computational Modelling

COMSOL Multiphysics (COMSOL, USA) was implemented for computational simulations of relevant transport phenomena. Simulations were performed in a simplified 2D-axisymmetric geometry given the spatial symmetry of the analyzed system (**Figure 4c**). To estimate the diffusion coefficient of 70 kDa dextran in the gel region, an AutoCAD file (Autodesk, USA) of the device geometry was imported and the model solved the diffusion equation:

$$\frac{\partial C}{\partial t} = D\frac{\partial^2 C}{\partial x^2} \tag{8}$$

where C is the molar concentration of the diluted molecule and *D* is the diffusion coefficient. Initial, boundary conditions of normalized concentrations $C_{max} = 1$ and $C_{min} = 0$ were applied at the left and right media channels, respectively. Zero flux conditions were imposed on the walls of the device. The diffusion coefficient was adjusted to match the experimental concentration profile at 2 hrs.

In an alternate model to investigate lymphatic drainage of interstitial solutes, a 2D-axisymmetric geometry was developed based on a lymphatic sprout within the gel region. The ascribed geometry of the sprout was based on the morphological characterization conducted in this study, while the gel domain extended to the full width of the center channel, and the height was based on the approximated distance between sprouts (~65 µm). An additional domain (0.5 µm thick) was implemented between the sprout and gel region that represented the lymphatic endothelium. To this domain, the Kedem-Katchalsky equation was imposed which governs the flux of solute (N) across a semipermeable membrane:

$$N = P_e\,\Delta C + (\Delta p_{EC}\,L_p)\cdot C\,(1-\sigma_f) \tag{9}$$

where the diffusive flux is driven by the concentration difference times the diffusive permeability, and the convective flux is given by the scalar product between the fluid velocity (obtained from the Starling equation) and the local concentration. The convective term also incorporates a filtration reflection coefficient (σ_f) which considers the fraction of solutes that permeate across the endothelium along with the

fluid flux. Fluid mechanics throughout the system was governed by the Brinkmann equation within the gel region and Stokes flow in nonporous regions. The transport of solutes throughout the rest of the system was governed by Fick's Second Law incorporating both diffusive and convective transport phenomena. The pressure and concentration boundary conditions were set to the same magnitudes as implemented in the experimental drainage assay with a constant value set at the inlet and a convective outflow boundary set at the outlet. Values for the intrinsic transport properties of the system (K, L_p, P_e) were determined experimentally in this work. Additional parameters such as the diffusion coefficient (D) and reflection coefficient (σ_f) for the different fluorescent solutes were based on previous studies from our lab. Given the high degree of similarity for the diffusion coefficient value corresponding to 70 kDa-dextran from our analysis to previous studies in our lab [1–3], we approximated the diffusion coefficients of the other fluorescent-dextrans from these studies. Additionally, from a previous study [4], a range of values for the reflection coefficient were considered in which the highest bound value ascribed to the lymphatic endothelium corresponded to that measured in blood microvascular networks given that the junctions of the lymphatics are exceedingly leakier. Transient numerical solutions were generated for a total computational time of 1000 seconds, similarly to the experimental assay (~15 min). A concentration probe was added at the end of the lymphatic sprout to measure the increase in concentration during drainage (**Figure S4**). Subsequently, we quantified the corresponding drainage rate. Additional simulations were performed to model drainage in decellularized samples, with the only corresponding difference being that the domain that represented the lymphatic endothelium was removed and all the aforementioned steps were then repeated. In additional experiments, we characterized the transport properties of our fibrin-based ECM before and after decellularization with the detergent solution (**Figure S10**). No significant changes appear in the transport properties of the ECM after washing with a 1% Triton X-100 solution.

To model the spatial distribution of chemotactic factors during the immune recruitment assay, we extended upon the preceding model, and incorporated an additional domain consisting of solid (impermeable) spheres embedded in the gel region which represented the migrating PBMCs. Transport equations were implemented based on our previous framework, and a reaction term was also incorporated, to account for the consumption of chemokines by the immune cells, based on similar studies [5,6] (R = 0.1 s^{-1} x C). Pressure boundary conditions were set at the same magnitude as the immune recruitment experiments to establish pathological interstitial flow. A zero-concentration boundary condition was imposed at the inlet and a convective outflow boundary at the outlet. Of special interest is the local secretion of chemokines by the lymphatics which was represented in the model as a constant flux condition at the endothelial domain. Despite not having experimental characterization of the secretion rates, we do have quantitative insight into the relative increase of secreted chemokines between the TNF-α-stimulated lymphatics and the unstimulated condition from the cytokine array analysis. For the diffusivity and endothelial transport

properties corresponding to the chemokines, an average value was implemented based on the range of molecular weights corresponding to the chemokines of interest (CCL21, CCL19, and CXCL12) [6]. A quasi-steady state assumption was implemented in the numerical solution. Given that the timescale for diffusive and convective phenomena is 10-100 orders of magnitude less than the timescale for cell migration across the gel region (at a speed of 1-10 μm/hr) [6,7], the spatial distribution of the chemokine reaches equilibrium prior to any cellular events. From this, we are able to obtain a generalized picture of the distribution of chemokines relative to the immune cells as they migrate through the system. A series of concentration probes were drawn across the embedded spheres, which rendered the concentration profile that the immune cells would encounter as they migrate through the gel towards the lymphatics. All parameters used for the simulations are listed in Table S2.

Scaling Analysis on Lymphatic Solute Drainage

Numerical results depict the uniform movement of solutes across the gel region as it approaches the lymphatic sprout. Once the solutes are located at the front end of the sprout, the pressure difference across the endothelium drives the entrance of solutes into the lumen compartment. It is at this region that the transport of solutes is accelerated, compared to the solutes that continue traveling within the gel region. This local increase in transport rate can be attributed to a lower hydraulic resistance exhibited by the lumen compartment (R_{lumen}), as compared to the resistance imposed by the gel region (R_{gel}) which can be validated on the basis of scaling (Equation S1):

$$\frac{R_{gel}}{R_{lumen}} \sim \frac{{\mu L}/{K r^2}}{{\mu L}/{r^4}} \sim \frac{r^2}{K} \qquad \text{S1}$$

where the same geometric parameters, length (L) and radius (r), are attributed to each region for direct comparison, with the dynamic viscosity (μ) and hydraulic permeability (K) also contributing to this estimation. Upon taking the ratio of resistances, we find that the scaling analysis reduces to a comparison in length scale where the hydraulic permeability is on the order of 10^{-13}, and the squared length of the radius is approximately 10^{-10} which results in a difference of 4 orders of magnitude. Thus, the lumen compartment provides a path of significantly less resistance where the solutes are preferentially transported, along with the fluid flow direction. Additionally, we can continue this analysis to verify the resistance contributed by lymphatic endothelium as Equation S2:

$$\frac{R_{endothelium}}{R_{lumen}} \sim \frac{{1}/{L_p r L}}{{\mu L}/{r^4}} \sim \frac{r^3}{L_p \mu L^2} \qquad \text{S2}$$

where the hydraulic conductivity (L_p) and the surface area of the sprout provide an estimate of the resistance to fluid passage through the endothelium. For different parameters corresponding to either the growth factor- or high flow-grown lymphatics, this ratio results in a value of either 0.7 or 0.3, respectively, which suggests that the endothelium does not act as a substantial barrier to fluid transport. Since the resistance by the lymphatic endothelium is comparable to that of the lumen, then both are negligible compared to the resistance imposed by the gel region. To further validate this, we also compared the hydraulic resistance between the gel and endothelium as Equation S3:

$$\frac{R_{gel}}{R_{endothelium}} \sim \frac{\mu L / K r^2}{1 / L_p r L} \sim \frac{L_p \mu L^2}{K r} \quad \text{S3}$$

from which we obtain a difference of at least 3 orders of magnitude, thus affirming that both resistances contributed by the lymphatic sprout (endothelial and luminal) are exceedingly lower than transport across the gel. Overall, these scaling arguments reveal that lymphatic sprouts facilitate solute drainage by providing a faster pathway for solute convection with minimal hindrance to fluid transport. This aligns with our previous experimental observations that 3D lymphatics achieve higher solute drainage rates, compared to a monolayer system.

To understand the underlying differences in transport phenomena between the lymphatic sprout and decellularized system, we further implemented scaling analysis with an emphasis on the relative timescales of solute transport within the lumen region. For this, we evaluated the Peclet number with appropriate adjustments to the scaling arguments accordingly to the studied system (Equation S4). For the lymphatic sprout model, the scaling parameters for the Peclet number follow as:

$$Pe_{lymph,\parallel} = \frac{(1-\sigma_f)u / L}{D / L^2} = \frac{(1-\sigma_f)u\, L}{D} \quad \text{S4}$$

where u indicates the average luminal velocity of the fluid, L corresponds to the sprout length, D continues to indicate the diffusion coefficient of the molecule and the reflection coefficient (σ_f) corrects for hindrance effects on the solutes. As we noted earlier, the timescale approximations in this analysis considers the relative competition of each phenomenon in the same, parallel direction. On a similar basis, the Peclet number for the decellularized sprout would be described as Equation S5:

$$Pe_{decell,\parallel} = \frac{u/_{L}}{D/_{L^2}} = \frac{u\,L}{D} \qquad \text{S5}$$

which simplifies the traditional Peclet number expression. For both of these parameters, the range of values is 15 to 47 for the lymphatic sprout model, and 24 to 77 for the decellularized system. Thus, the convective flux of solutes dominates their luminal transport, for both systems, which is in line with our previous analysis that this fluid pathway provides a faster route for solute drainage. However, diffusion of solutes simultaneously occurs in the lateral/radial direction which is responsible for the solute leakage from the lumen into the gel region observed in our computational results. As such, the Peclet number for this analysis (Equation S6) would consider the diffusive transport timescale in the radial direction as:

$$Pe_{decell,\perp} = \frac{u/_{L}}{D/_{r^2}} = \frac{u\,r^2}{D\,L} \qquad \text{S6}$$

for the decellularized system. Similarly, modifying the scaling arguments (Equation S7) for the diffusive rate in the lymphatic sprout yields:

$$Pe_{lymph,\perp} = \frac{(1-\sigma_f)\,u/_{L}}{P/_{r}} = \frac{(1-\sigma_f)\,u\,r}{P\,L} \qquad \text{S7}$$

where *P* is the diffusive permeability of the endothelium. Taking this new parameterization for the relative transport rate, we calculated that the Peclet numbers are in the range of 0.02 to 0.06, for the decellularized system, and 1.2 to 7, for the lymphatic sprout model, which implies that the diffusive rate by which these solutes are leaking out of the lumen has a significant contribution in the overall drainage in both model systems. However, the presence of the lymphatic endothelium dampens the relative magnitude of this diffusive leakage, as demonstrated in the simulation results and scaling analysis. Thus, enhancing the solute drainage rates compared to bare, empty channels which are also consistent with the normalized drainage measurements presented in the previous experimental section.

Blood-to-Lymphatic Protein Transport Analysis

We start this analysis by defining a simplified system that would model the tissue interstitium. Based on current microfluidic designs, the on-chip system integrates each vascular model at different compartments with a central gel region acting as the scaffold that bridges the two vasculatures (**Figure S11**). As such, we can restrict our analysis to this gel region as a one-dimensional system and consider the transport contribution of each vascular compartment as a flux boundary condition.

The fluid transport across this gel system can be described on the basis of Darcy's law, discussed earlier in this work as:

$$u = \frac{k}{\mu}\frac{\left(p_{g,o} - p_{g,w}\right)}{w}$$

where $p_{g,o}$ and $p_{g,i}$ indicate pressures at the initial, blood vascular interface boundary and opposite, lymphatic outlet boundary, respectively, while k represents the hydraulic permeability (previously characterized) and u corresponds to the average interstitial fluid velocity. Additionally, to account for the fluid flux from each vascular compartment, a boundary condition is applied at each adjacent side of the gel region where the fluid flux is given by the Starling equation:

$$u = \frac{A_{s,B}H}{V_{T,B}}L_{p_B}\left(p_B - p_{g,o}\right) = \frac{A_{s,L}H}{V_{T,L}}L_{p_L}\left(p_{g,w} - p_L\right)$$

where L_p and A_s/V_T correspond to the hydraulic conductivity and vascular surface area per unit tissue volume, respectively. Both parameters have distinctive values corresponding to the particular vascular system, blood (B) or lymphatic (L). Given that oncotic-driven pressure differences are negligible in our *in vitro* systems, hydraulic pressure differences across each vascular compartment determine the rate of fluid transport. For this, we consider a uniform intravascular pressure distribution for the blood (p_B) and lymphatic (p_L) vasculatures. Finally, the length of this boundary (H) is multiplied into the equation to obtain the resultant fluid velocity at each boundary. By applying mass conservation and incompressibility, we can establish that all three independent equations described above, relate to the same fluid velocity. Since all the transport and geometric parameters have been previously characterized, we can solve the corresponding equations to obtain the pressure distribution necessary to impose a physiological interstitial fluid velocity of 1 μm/s.

Despite having a total of four unknown pressures, we can consider the fluid pressure at the lymphatic vasculature as the zero-pressure reference, thus allowing us to solve for the remaining pressures. A MATLAB algorithm was generated to solve the system of equations for the desired pressures. From the solution, we find that by pressurizing the blood microvasculature to 500 Pa relative to the lymphatic outlet, we are able to establish physiological interstitial fluid flow exiting the blood compartment and drained by the lymphatics. Previous work by our lab has achieved this experimental setup in our on-chip microvascular networks.

We next consider the protein transport within this interstitial space to which we apply the non-dimensional mass conservation equation for a steady state condition in a uniform velocity field and neglecting uptake or degradation within the computational domain:

$$0 = \frac{D}{u\,w}\frac{\partial^2 \hat{C}}{\partial \hat{x}^2} - \frac{\partial \hat{C}}{\partial \hat{x}} = \frac{1}{Pe}\frac{\partial^2 \hat{C}}{\partial \hat{x}^2} - \frac{\partial \hat{C}}{\partial \hat{x}}$$

where the Peclet number (*Pe*) naturally arises from the dimensional analysis of the equation and allows us to simplify our analysis by weighting the dominant transport mode. On a similar basis to our previous fluid transport analysis, the relative contribution of mass transport by the blood and lymphatic vasculatures are taken into account with boundary flux conditions. For the blood compartment, the rate at which extruded proteins enter the gel region is given by:

$$N_B = \frac{A_{s,B} H}{V_{T,B}} P_B\left(C_B - C_{g,o}\right) + u\left(1 - \sigma_{f,B}\right) C_B$$

where the diffusive flux is given by the local difference in concentration between the blood compartment (C_B) and the adjacent gel periphery ($C_{g,o}$) times the endothelial permeability (P_B) and the effective endothelial surface area. The convective flux contribution is the product of the local fluid velocity and the solute concentration exiting the compartment while considering reflection effects ($\sigma_{f,B}$) through the endothelium. Accounting for the flux at the lymphatic compartment, we implemented an outflow boundary where the convective flux dominates the transport of proteins through the lymphatics given the results of our scaling analysis in the previous section. As such, the flux equation simplifies to:

$$N_L = u\left(1 - \sigma_{f,L}\right) C_{g,w}$$

where the local concentration at the adjacent lymphatic outlet ($C_{g,o}$) is transported through the lymphatics by convection with filtration effects ($\sigma_{f,L}$) included. For this analysis, we are interested in predicting the ratio of concentrations between the steady protein content in the interstitial space relative to the blood intravascular concentration (C_g/C_B). This allows us to compare the steady state concentration distribution in our *in vitro* system to that of *in vivo* and clinical measurements where this normalized concentration ratio is often implemented. As a model protein, we considered the specific case of albumin transport in our engineered vascular systems. Given our prior characterization on the transport properties of this protein in both our blood and lymphatic microvascular systems, we can directly apply our measured values to this

analysis. We first simplify our calculation by considering that the convective transport of albumin would dominate its steady state distribution ($Pe \sim 10$), thus a uniform concentration within the interstitial matrix would follow. Then, we consider that the equilibrium interstitial concentration is determined by the balanced flux of each vascular compartment as:

$$N_B = N_L \,,$$

$$\frac{A_{s,B} H}{V_{T,B}} P_B \left(C_B - C_g\right) + u\left(1 - \sigma_{f,B}\right) C_B = u\left(1 - \sigma_{f,L}\right) C_g$$

from which we can directly solve for the normalized concentration (C_g/C_B). We solve for this mass transport analysis with our MATLAB algorithm which indicated that the average concentration of albumin in the interstitial region would be 0.32x that in the blood intravascular compartment. We validated this estimation by implementing a COMSOL-based model to obtain a numerical solution to this transport analysis. In line with our calculations, the computational results indicated a uniform concentration distribution in the interstitial region which resulted to be at a value of 0.31x to that of the blood intravascular concentration. This value is well within the reported ranges for clinical assessments of plasma proteins (0.3 - 0.4)[8]. Thus, the integration of the lymphatic vascular platform, described in this work, with our blood microvascular system has the potential to recapitulate the homeostatic, equilibrium concentration of proteins across the interstitial matrix.

References

1. Funamoto, K., Yoshino, D., Matsubara, K., Zervantonakis, I.K., Funamoto, K., Nakayama, M., Masamune, J., Kimura, Y., and Kamm, R.D. (2017) Endothelial monolayer permeability under controlled oxygen tension. *Integr. Biol.*, **9** (6), 529–538.
2. Uzel, S.G.M., Amadi, O.C., Pearl, T.M., Lee, R.T., So, P.T.C., and Kamm, R.D. (2016) Simultaneous or Sequential Orthogonal Gradient Formation in a 3D Cell Culture Microfluidic Platform. *Small*, **12** (5), 612–622.
3. Zervantonakis, I.K., Hughes-Alford, S.K., Charest, J.L., Condeelis, J.S., Gertler, F.B., and Kamm, R.D. (2012) Three-dimensional microfluidic model for tumor cell intravasation and endothelial barrier function. *Proc. Natl. Acad. Sci. U. S. A.*, **109** (34), 13515–13520.
4. Offeddu, G.S., Possenti, L., Loessberg-Zahl, J.T., Zunino, P., Roberts, J., Han, X., Hickman, D., Knutson, C.G., and Kamm, R.D. (2019) Application of Transmural Flow Across In Vitro Microvasculature Enables Direct Sampling of Interstitial Therapeutic Molecule Distribution. *Small*, **15** (46), 1902393.

5. Polacheck, W.J., Charest, J.L., and Kamm, R.D. (2011) Interstitial flow influences direction of tumor cell migration through competing mechanisms. *Proc. Natl. Acad. Sci. U. S. A.*, **108** (27), 11115–11120.
6. Fleury, M.E., Boardman, K.C., and Swartz, M.A. (2006) Autologous morphogen gradients by subtle interstitial flow and matrix interactions. *Biophys. J.*, **91** (1), 113–121.
7. Li, R., Serrano, J.C., Xing, H., Lee, T.A., Azizgolshani, H., Zaman, M., and Kamm, R.D. (2018) Interstitial flow promotes macrophage polarization toward an M2 phenotype. *Mol. Biol. Cell*, **29** (16), 1927–1940.
8. Rutili, G., and Arfors, K. -E (1977) Protein Concentration in Interstitial and Lymphatic Fluids from the Subcutaneous Tissue. *Acta Physiol. Scand.*, **99** (1), 1–8.

Supplementary Tables

Table S1: Experimental and computational default parameters for lymphatic drainage model.

Parameter	Symbol	Value
Source concentration of 3 kDa dextran	$c_{o,\ 3kDa}$	3.3 x 10^{-2} mol/m^3
Source concentration of 10 kDa dextran	$c_{o,\ 10kDa}$	1.0 x 10^{-3} mol/m^3
Source concentration of 70 kDa dextran	$c_{o,\ 70kDa}$	1.4 x 10^{-3} mol/m^3
Diffusion coefficient of 3kDa dextran	D_{3kDa}	14.5 x 10^{-11} m^2/s
Diffusion coefficient of 10 kDa dextran	D_{10kDa}	9 x 10^{-11} m^2/s
Diffusion coefficient of 70 kDa dextran	D_{70kDa}	4.5 x 10^{-11} m^2/s
Hydraulic conductivity of monolayer lymphatics	$L_{p\ M}$	3.9 x 10^{-6} m/Pa s
3 kDa dextran permeability in monolayer lymphatics	$P_{M,\ 3kDa}$	7.0 x 10^{-8} m/s
10 kDa dextran permeability in monolayer lymphatics	$P_{M,\ 10kDa}$	1.3 x 10^{-8} m/s
70 kDa dextran permeability in monolayer lymphatics	$P_{M,\ 70kDa}$	1.2 x 10^{-8} m/s
Hydraulic conductivity of growth factor-grown lymphatics	$L_{p\ GF}$	3.6 x 10^{-6} m/Pa s
3 kDa dextran permeability in growth factor-grown lymphatics	$P_{GF,\ 3kDa}$	2.3 x 10^{-8} m/s
10 kDa dextran permeability in growth factor-grown lymphatics	$P_{GF,\ 10kDa}$	4.3 x 10^{-9} m/s
70 kDa dextran permeability in growth factor-grown lymphatics	$P_{GF,\ 70kDa}$	3.8 x 10^{-9} m/s
Hydraulic conductivity of high flow-grown lymphatics	$L_{p\ HF}$	8.1 x 10^{-6} m/Pa s
3 kDa dextran permeability in high flow-grown lymphatics	$P_{HF,\ 3kDa}$	7.2 x 10^{-8} m/s
10 kDa dextran permeability in high flow-grown lymphatics	$P_{HF,\ 10kDa}$	3.0 x 10^{-8} m/s
70 kDa dextran permeability in high flow-grown lymphatics	$P_{HF,\ 70kDa}$	2.4 x 10^{-8} m/s
Reflection coefficient of 3 kDa dextran	$\sigma_{f,\ 3kDa}$	0.2-0.4
Reflection coefficient of 10 kDa dextran	$\sigma_{f,\ 10kDa}$	0.4-0.8
Reflection coefficient of 70 kDa dextran	$\sigma_{f,\ 70kDa}$	0.4-0.8

Table 2: Experimental and computational default parameters for chemokine transport model.

Diffusion coefficient of chemokines	D_{ch}	14.5 x 10^{-11} m^2/s
Flux of chemokines from the lymphatics	N_{ch}	1 x 10^{-6} – 2.5 x 10^{-6} mol/m^2 s
Hydraulic conductivity of high flow-grown lymphatics	$L_{p\ HF}$	8.1 x 10^{-6} m/Pa s
Chemokine permeability in high flow-grown lymphatics	$P_{HF,\ ch}$	7 x 10^{-8} m/s
Reflection coefficient of chemokines	$\sigma_{f,\ ch}$	0.2
Consumption rate of chemokines by PBMCs	R_{ch}	0.5 x C mol/s

Supplementary Figures

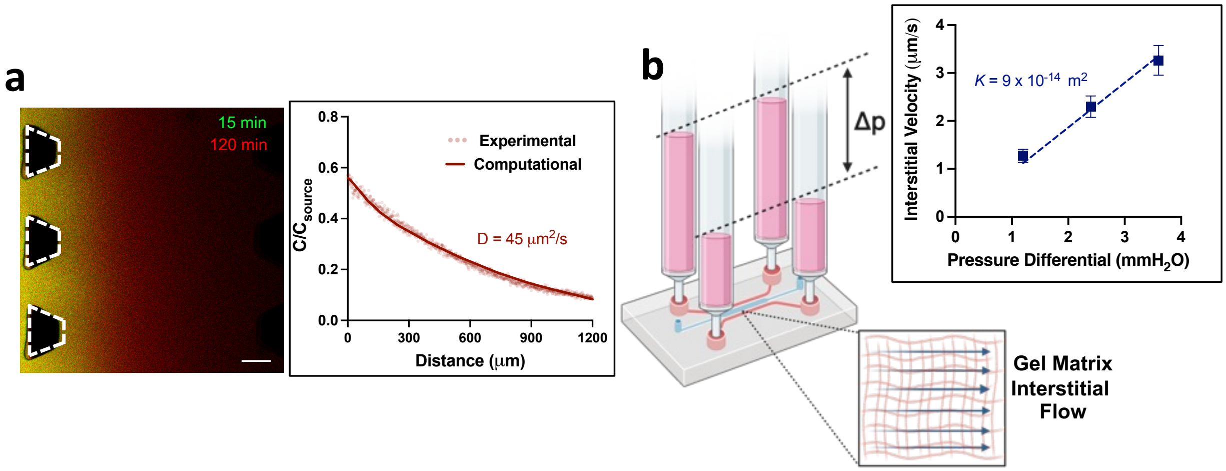

Figure S1. a) Representative image of the diffusion of fluorescently-labeled dextran in the microfluidic device for time intervals of 15 min (green channel) and 120 min (red channel). Scale bar is 100 µm. Intensity-concentration profile for the experimental and computational data at 120 min from which we estimated the coefficient of diffusion (D). b) Schematic representation of the interstitial flow set up, where a hydrostatic pressure difference (Δp) drives interstitial flow across the gel matrix compartment. Interstitial flow velocity as a function of the pressure differential. The linear fit applied is based on Darcy's law to extract the hydraulic permeability (K) of the gel. Data shown corresponds to the mean ± S.E.M., n=5 samples (microfluidic devices) per condition, and m=3 ROI images per sample.

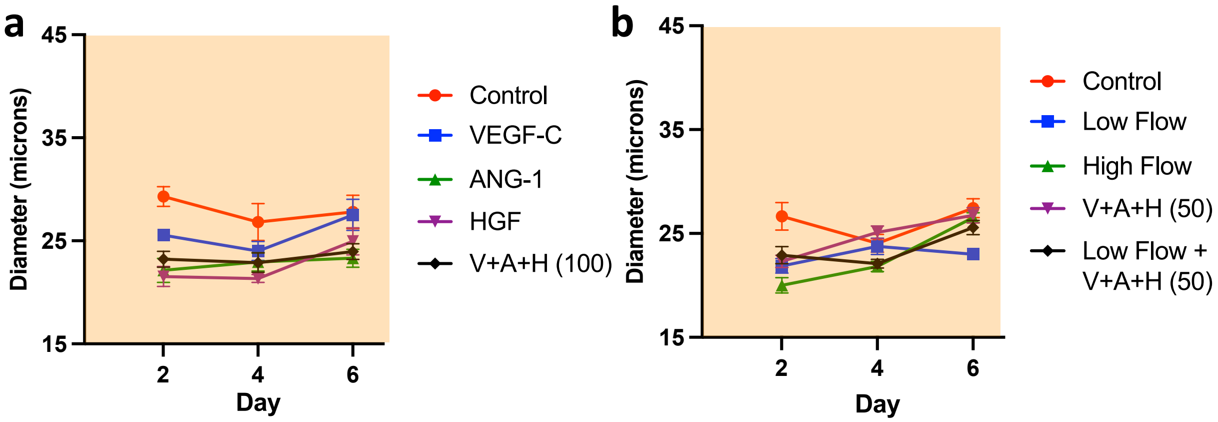

Figure S2. Quantitative analysis of lymphatic vessel diameters for a) biochemical-stimulated lymphatics with growth factors and b) interstitial flow-stimulated lymphatics. Highlighted regions correspond to *in vivo* values. Data shown corresponds to the mean ± S.E.M., n=3 samples (microfluidic devices) per condition, and m=9 ROI images per sample. Highlighted regions correspond to *in vivo* values.

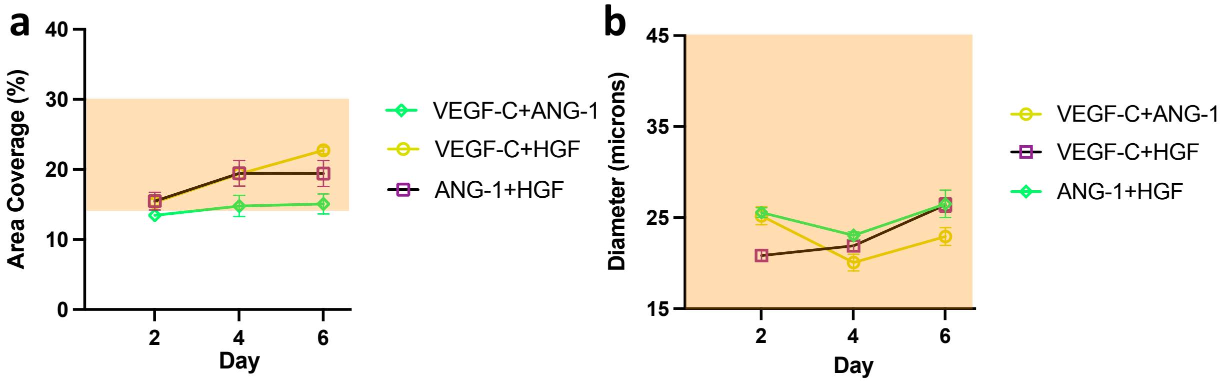

Figure S3. Quantitative analysis of lymphatic vessel morphology: a) lymphatic area of coverage and b) lymphatic vessel diameter for additional combinations of growth factors. Highlighted regions correspond to *in vivo* values. Data shown corresponds to the mean ± S.E.M., n=3 samples (microfluidic devices) per condition, and m=9 ROI images per sample. Highlighted regions correspond to *in vivo* values.

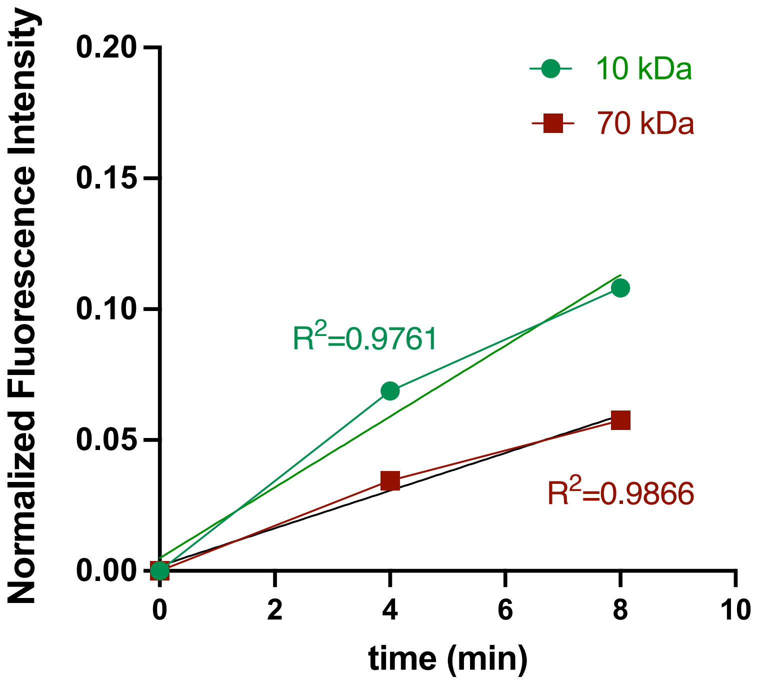

Figure S4. Temporal plot of fluorescence intensity corresponding to solute drainage measurements of 10 kDa dextran for high-flow engineered lymphatics. Measurements begin at the time the fluorescence intensity in the measurement region first begins to rise, roughly corresponding to time at which the concentration front reaches the upstream end of the lymphatic network.

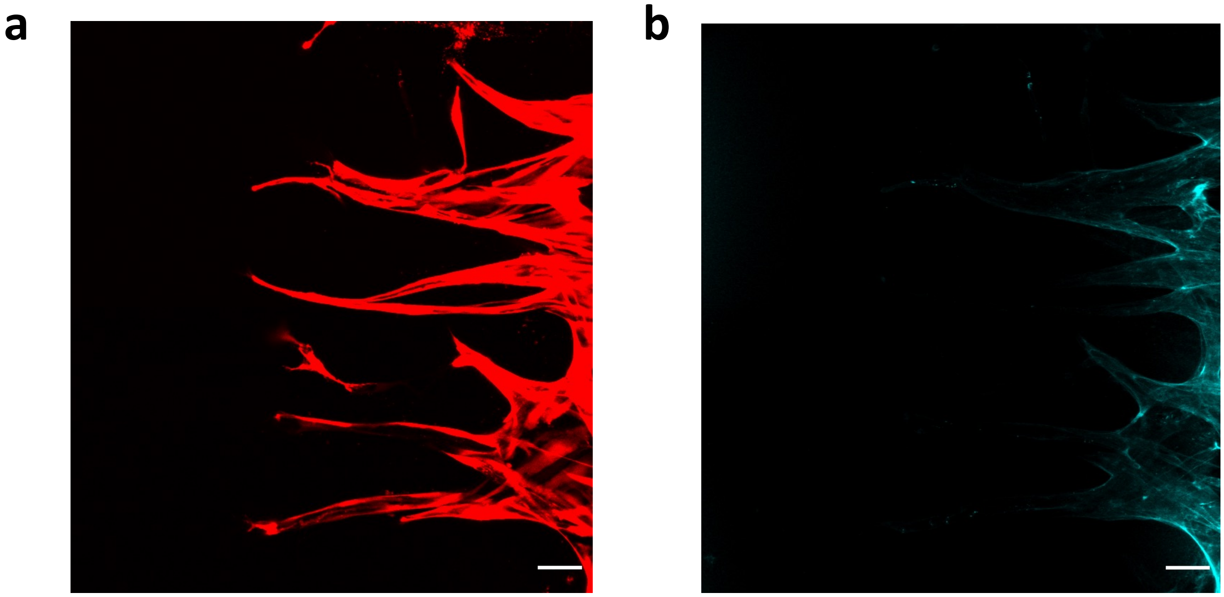

Figure S5. Representative images of the engineered lymphatic microvasculature where lymphatic endothelial cells express RFP a) are also stained with lectin b) to stain the glycocalyx. All scale bars are 50 μm.

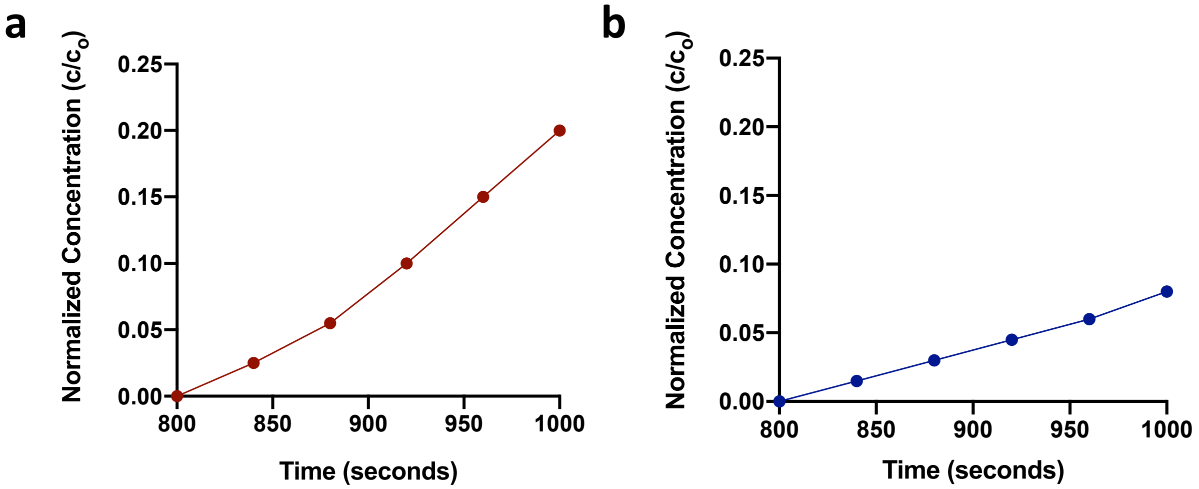

Figure S6: Concentration plot corresponding to a concentration probe placed at the end of the lumen compartment, thus measuring the increase in solute concentration during drainage by the lymphatic sprout model a) and the decellularized-sprout system b). Both plots pertain to 10 kDa dextran-based solute properties.

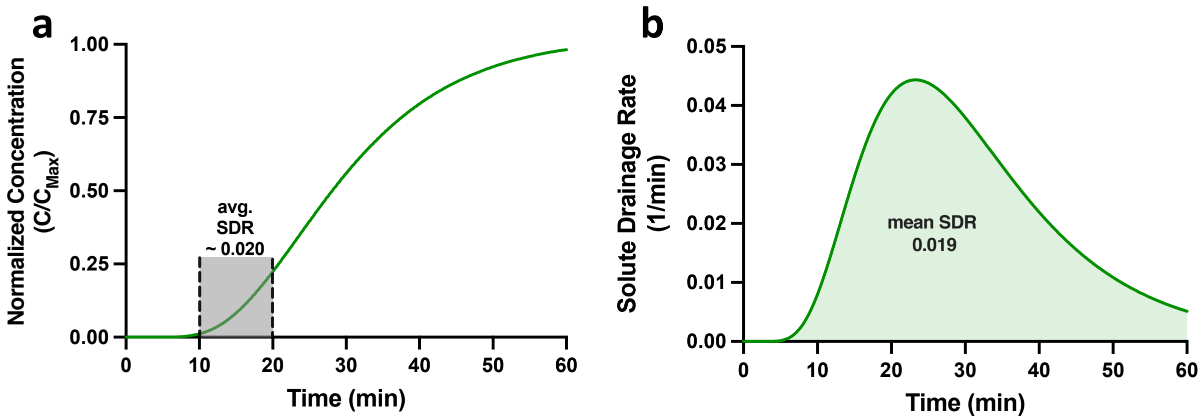

Figure S7: (a) Simulation results monitoring the increase in concentration in the outlet until reaching saturation. (b) Variation in solute drainage rate corresponding to slope fluctuations from the concentration plot (a). Estimating the overall solute drainage rate by the average rates in the initial 10 minutes (a), provides a comparable value to estimating the exact mean from the overall variation plot (b). Simulations pertain to 10 kDa dextran-based solute and high flow-engineered lymphatic properties

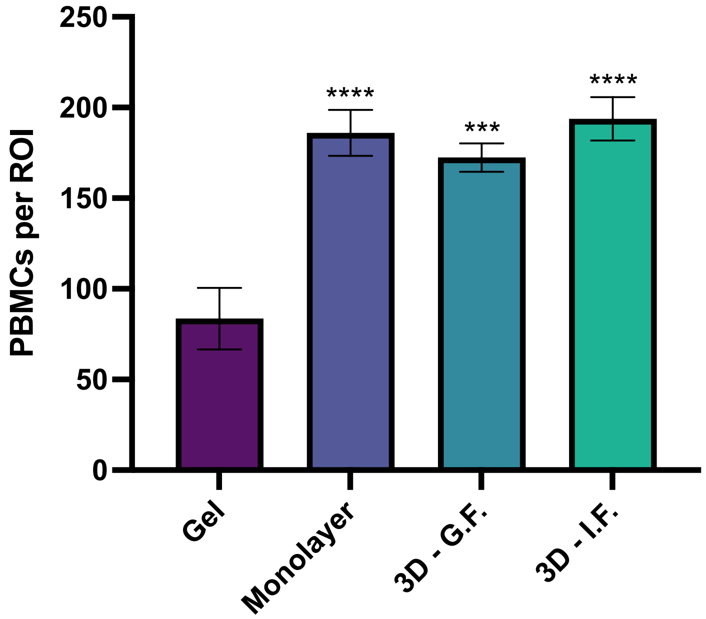

Figure S8. Quantitative analysis from the PBMCs infiltration assay performed on varying experimental conditions as described in the plot. Statistical significance is reported with respect to the gel sample. Data shown corresponds to the mean ± S.E.M., n=4 samples (microfluidic devices) per condition, and m=3 ROI images per sample. Statistical significance system significance is reported with respect to the gel condition, where ***$p<0.001$ and ****$p<0.0001$ (based on the one-way ANOVA test).

	L	C	P
**IgG**	0.64	0.41	0.46
**CCR7**	0.05	0.06	0.51
**CXCR4**	0.02	0.01	0.70
**CCR7 + CXCR4**	0.03	0.12	0.58

Figure S9. Heat map of PBMC distribution within the gel region for specified antibody-neutralization conditions by each row, and locations by column. The table columns for L, C and P correspond to the gel region with lymphatics, central gel region and region where the PBMCs are introduced, respectively.

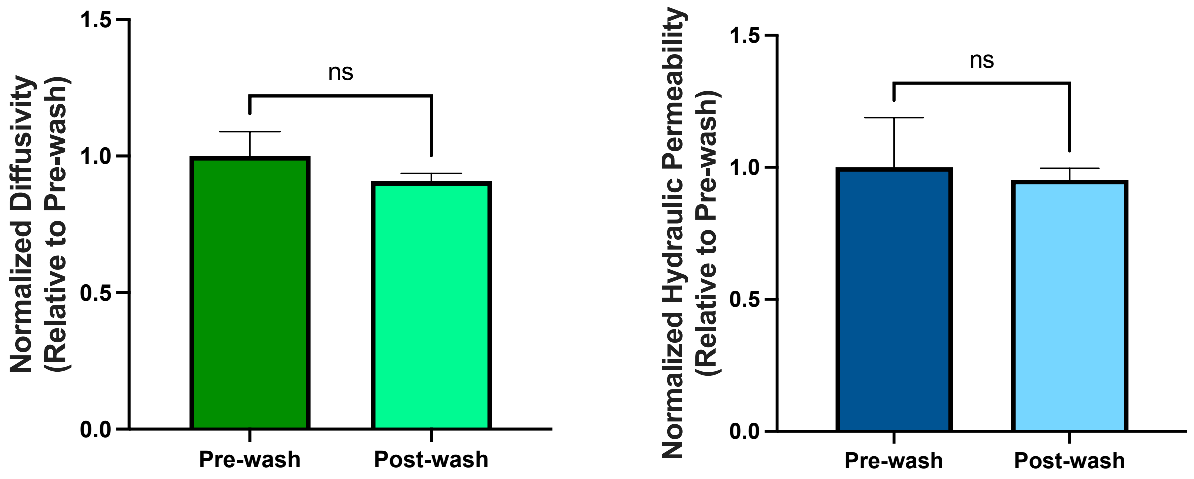

Figure S10: Transport properties characterization of microfluidic-compartmentalized fibrin matrices before and after washing with a 1% Triton X-100 detergent solution. Data shown corresponds to the mean ± S.E.M., n=2 samples (microfluidic devices) per condition, and m=3 ROI images per sample. No significant differences are observed across conditions.

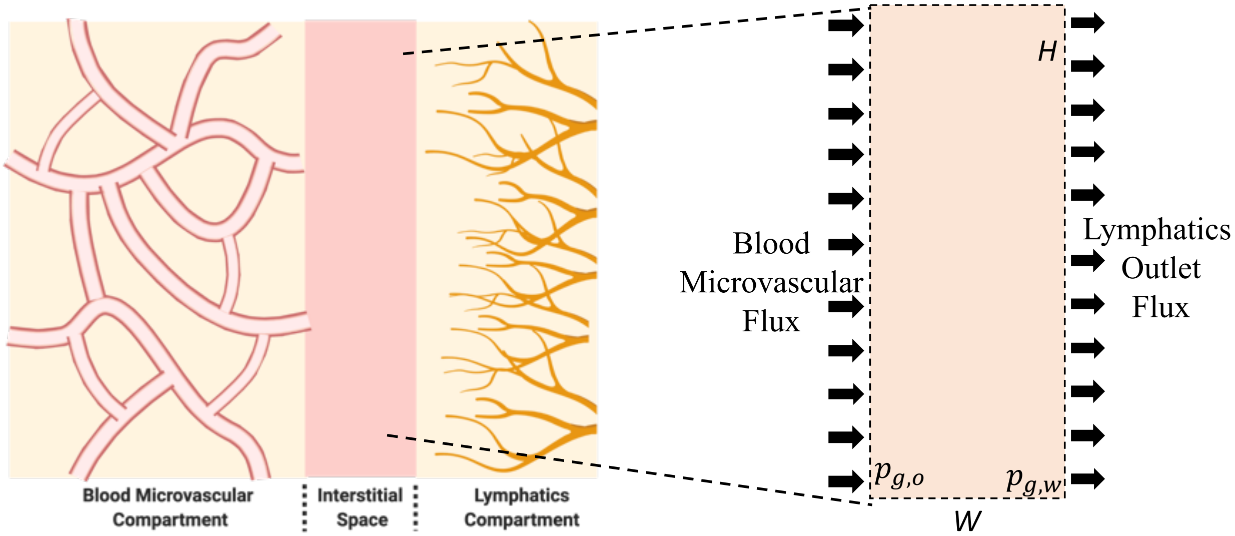

Figure S11: Schematic representation of the on-chip integration of the blood and lymphatic microvascular platforms (left) and the interstitial space region taken as the control volume (right).
